# Combining genomic and network characteristics for extended capability in predicting synergistic drugs for cancer

**DOI:** 10.1038/ncomms9481

**Published:** 2015-09-28

**Authors:** Yi Sun, Zhen Sheng, Chao Ma, Kailin Tang, Ruixin Zhu, Zhuanbin Wu, Ruling Shen, Jun Feng, Dingfeng Wu, Danyi Huang, Dandan Huang, Jian Fei, Qi Liu, Zhiwei Cao

**Affiliations:** 1 School of Life Sciences and Technology, Tongji University, Shanghai 200092, China; 2 Shanghai Research Center for Model Organisms, Shanghai 200092, China

## Abstract

The identification of synergistic chemotherapeutic agents from a large pool of candidates is highly challenging. Here, we present a Ranking-system of Anti-Cancer Synergy (RACS) that combines features of targeting networks and transcriptomic profiles, and validate it on three types of cancer. Using data on human β-cell lymphoma from the Dialogue for Reverse Engineering Assessments and Methods consortium we show a probability concordance of 0.78 compared with 0.61 obtained with the previous best algorithm. We confirm 63.6% of our breast cancer predictions through experiment and literature, including four strong synergistic pairs. Further *in vivo* screening in a zebrafish MCF7 xenograft model confirms one prediction with strong synergy and low toxicity. Validation using A549 lung cancer cells shows similar results. Thus, RACS can significantly improve drug synergy prediction and markedly reduce the experimental prescreening of existing drugs for repurposing to cancer treatment, although the molecular mechanism underlying particular interactions remains unknown.

## Abstract (web summary)

Predicting combinations of chemotherapeutic drugs that act synergistically is challenging. Here the authors take a computational approach to predict synergistic pairs, validate novel pairs using several cancer cell lines, and assess toxicity in a zebrafish xenograft model.

## Graphical abstract

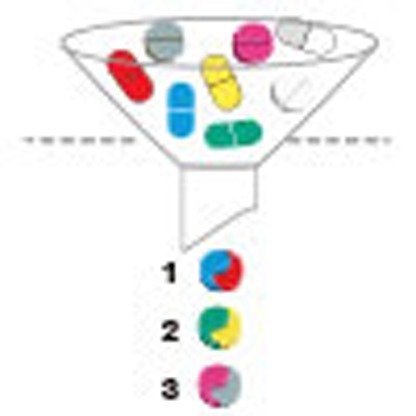

Cancer is a complex disease involving multiple factors and pathways. In the clinic, treatment with anticancer drugs through monotherapy has been frequently associated with acquired resistance and side effects for a couple of years, whereas combinational therapy has been widely explored as a better alternative^1^. In recent years, particular attention has been paid to synergistic drugs, which exhibit a greater overall therapeutic effect than the sum of the individual effects and which present largely reduced side effects because of the lower dosage of each ingredient compared with that used in monotherapy^2^. To facilitate the identification of potential synergistic agents, high-throughput screening platforms have been established with hundreds of matrix blocks designed for testing different concentrations of each drug on various cell lines before subsequent animal or clinical tests^3^,^4^,^5^. Thus, *in silico* computational methods are fully expected to complement laborious experiments and accelerate screening for the identification of candidates with desirable effects on specified cancers.

Mathematical models based on ordinary differential equations have been successfully applied to the quantitative calculation of cellular responses to drug intervention targeting specific networks^6^. However, the application to cancer has been largely restricted due to the difficulty in determining the dynamic parameters of large cancer networks (CNs). Consequently, without sufficient reaction parameters, alternative computational algorithms have to be developed to accelerate the screening of synergistic drugs. Zhao *et al*.^7^ recently proposed a useful method for predicting synergistic drug effects based on transcriptomic profiles. Subsequently, the DrugComboRanker platform was built based on the hypothesis that synergistic drugs may target different signaling modules of the disease network. Although promising, the few published models lack rigorous experimental validation and are not generalizable to arbitrary compound combinations^8^. To promote the development of *in-silico* methods for computing drug synergy, the Dialogue for Reverse Engineering Assessments and Methods (DREAM) consortium launched an international open challenge for the development of computational models that can be used to objectively and systematically evaluate the accuracy and specificity of drug synergy predictions. Despite a complete lack of publications and established methodologies in this research area, 31 individual methods from >13 countries were entered in this challenge. Experimental data on drug synergy were collected for 91 binary pairs derived from 14 compounds applied to the human diffuse large B-cell lymphoma (DLBCL) cell line OCI-LY3, and these data were complemented with information of the gene expression profiles of the cells perturbed with these individual compounds. Among the 31 methods, only three techniques performed significantly better than random chance^8^, suggesting that despite the availability of different source of information, the overall accuracy of prediction of synergistic drugs remains a significant challenge. Specifically, the current models are either based on intuitive hypotheses or designed for synergistic drugs for the treatment of all types of diseases but have been validated under limited conditions. However, drug synergy has emerged as strongly context-dependent, likely reflecting the effects on not only a specific disease but also testing platforms for different cell lines or even drug dosages^8^. Although promising, hypothesis-driven, rather than data-driven, models may identify only partially synergistic mechanisms, whereas other models generalized for all diseases may miss some significant characteristics contributing to drug synergy in specific diseases, which would likely reduce their performance when applied to a specific disease, such as cancer. Indeed, the best method reported by DREAM obtained an overall probability concordance-index (PC-index) of 0.613, compared with a PC-index of 0.50 obtained from random chance and a ground truth PC-index of 0.90, whereas Zhao's method only achieved a PC-index of 0.575 (ref. 8). Using The Cancer Genome Atlas (TCGA) data on lung adenocarcinoma and endocrine receptor (ER)-positive breast cancer, 28 to 38% of the combinations predicted using DrugComboRanker showed evidence of positive effects consistent with the published literature^9^. These results suggest that there remains a large gap between the power of computational prediction and experimental validation. Thus, continuing efforts to develop new methods to predict compound synergy based on empirical data and experimental validation are needed.

At present, a number of synergistic drug combinations have been accumulated in databases or the literatures in the anticancer field^10^. In addition, knowledge of the molecular mechanisms of existing synergistic drugs has been partially summarized from different perspectives. For example, synergistic drugs may target multiple points in a pathway and its cross-talked pathways^11^. Compensatory pathway interactions and adaptive resistance, as well as molecular and pharmacological data^12^ and gene expression similarities^13^ have also been suggested^3^. On the basis of previous studies, the identification of additional synergistic drugs among potential candidates is possible when useful clues can be formulated into a prediction model.

In this manuscript, we propose a model for the prediction of synergistic drug combinations specifically for the treatment of cancer. In the case of anticancer therapy, only a limited number of synergistic drugs have been identified, but the combinations between these drugs remain largely unknown or unexplored. To manage this severe data imbalance, we establish a semi-supervised learning model called Ranking-system of Anti-Cancer Synergy (RACS) to address the limited positive/labelled samples and the large set of unknown/unlabelled combinations. The drug pharmacological characteristics, drug targeting networks and transcriptomic profiles are initially tested to differentiate known synergistic combinations among a set of unlabelled data. Subsequently, the significant parameters are further formulated into RACS to calculate the synergistic potential of these drug combinations. The RACS results are extensively validated via experiments on DREAM data of the human β-cell lymphoma cell line OCI-LY3, the breast cancer cell line MCF7 and the human lung adenocarcinoma cell line A549. *In vivo* validation of the synergistic effects and potential toxicity is also examined using a zebrafish-based human cancer cell xenograft model. The framework of RACS can effectively improve drug synergy prediction for guiding experimental searching despite of the unclear synergistic mechanism.

## Results

### RACS to predict synergistic potential of drug combinations

**Figure 1:**
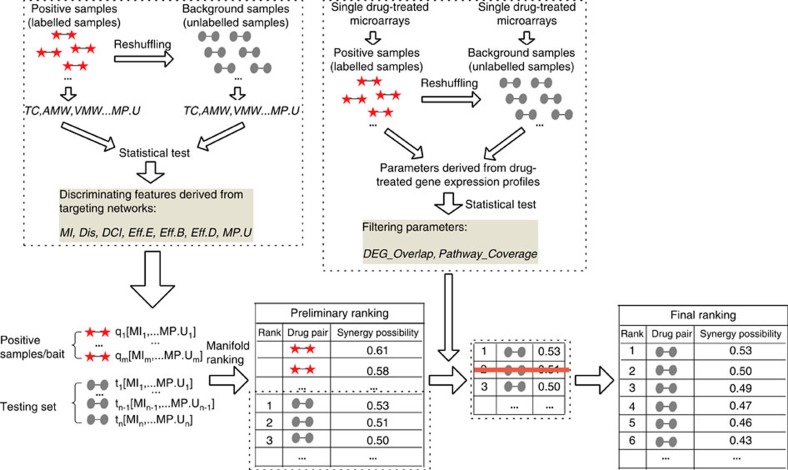
Workflow for RACS. The pairs of red stars denote the known synergistic drug pairs (labelled pairs), while the pairs of gray ellipses denote the unlabelled pairs. For any testing drugs provided by users, the household positive pairs are incorporated as baits into the data set. All the bait pairs and unlabelled testing pairs will be ranked via manifold-ranking method in the space of multiple features. After removing the baits, the preliminary ranking will be further refined by the gene expression filters.

RACS was developed to predict the synergistic potential of the available anticancer drugs. Given a limited number of known synergistic combinations, RACS ranks drug pairs according to similarities with known samples in a specified multi-feature space. To achieve flexibility for compounds without sufficient information, RACS is performed in two steps (Fig. 1): (1) Preliminary ranking: RACS computes the synergistic potential for queried drug pairs in terms of similarities to known/labelled pairs relative to a targeted biological network; and (2) Secondary filtering: The preliminary ranking is further refined based on functional correlations between individual drugs by examining the gene expression profiles of tested cell lines.

A total of 26 pairs of known synergistic anticancer drugs among 33 tested drugs were collected as positive/labelled samples with corresponding drug targets and transcriptomic profiles of single-drug treatment on specific cell lines. The reshuffling of the 33 drugs constitutes 502 pairs of unlabelled samples. Fourteen features covering the chemical structure, pharmacology and functional and network properties of the drug targets were noted (Supplementary Table 1), but, only seven features were identified as significantly different between the synergistic and unlabelled pairs (Table 1). These seven features were subsequently selected to formulate RACS. Similarly, five parameters describing correlations between differentially expressed genes (DEGs) were tested (Supplementary Table 2), and two parameters were significantly different between the positive and unlabelled samples (Supplementary Table 3). These two parameters were used as further filters to improve the preliminary ranking.

All of the labelled samples and unlabelled tested pairs were represented through the seven significant features for preliminary ranking. Subsequently, a semi-supervised learning method incorporating a manifold ranking technique was applied to enrich the labelled/bait pairs at the top of the ranked list^14^. The order of queried pairs in the optimized ranking list was considered the preliminary ranking. Moreover, the transcriptomic features were applied to filter out drugs pairs without significant functional correlation.

### Significant improvement on DREAM data of DLBCL cells

**Figure 2:**
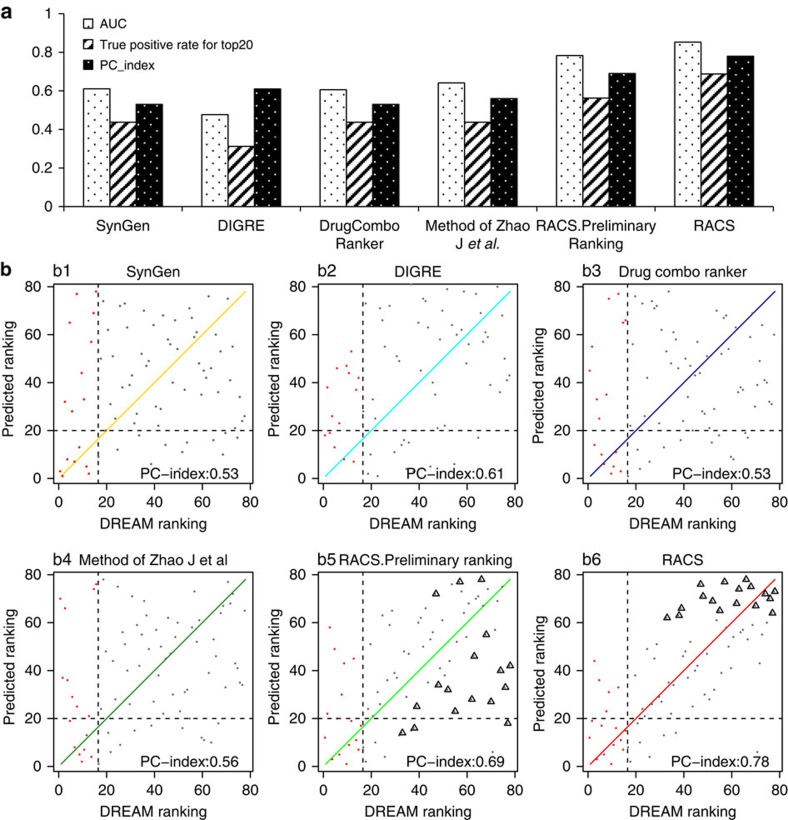
Performance of RACS on human β-cell lymphoma cell line OCI-LY3 data. (**a**) Performance comparison of different ranking methods. White bar with black points: barplot for AUC metric. White bar with black slashes: barplot for true positive rate for the top 20 predicted pairs. Black bar with white points: barplot for PC-index. (**b**) Detailed ranking agreement between DREAM and computational models. The red dots are true synergistic drug combinations, while the grey dots are the non-synergistic ones confirmed from DREAM experiments. The desired models would be able to map dots into the diagonal line as much as possible. The vertical black dashed lines indicate the boundary between the top 16 synergistic pairs and non-synergistic ones, while the horizontal black dashed line illustrates boundary between the top 20 predicted ranking and the rest 58 ones. b5-b6: The dots with triangle are those pairs being filtered out to down-list from the preliminary ranking in RACS.

To evaluate its prediction capacity, we applied RACS to the standard data obtained from the DREAM consortium^8^. The experimental data on the combinational activity of these drugs on the human diffuse large B-cell lymphoma (DLBCL) cell line OCI-LY3 together with the gene expression profiles upon drug treatment at different times and under different concentrations were reviewed to determine the effects of the binary combinations of 14 distinct drugs/compounds. Because mitomycin C is a potent DNA crosslinker and its targets have not been clearly specified^15^, 78 drug pairs among 13 agents were tested as unlabelled samples using RACS (Supplementary Table 4). The performances of peering methods, including DIGRE (the best-performing method included in the DREAM report), SynGen (a method explicitly proposed by the DREAM organizers), and additional online methods, including DrugComboRanker and Zhao's method, were examined using the same set of data. An extensive comparison of the models using DREAM data was performed based on the the area under the curve (AUC) value, the true positive rate, and the proposed PC-index, as shown in Fig. 2a.

RACS obtained a PC-index of 0.78 with an AUC value of 0.85, indicating that this method displayed the best performance in various tests. To further investigate the ability of each method to predict synergy using DREAM data, the agreement between the DREAM ranking and the predicted ranking was mapped for each drug pair and each method, as shown in Fig. 2b. A higher number of dots that aggregated into a diagonal line indicated a better prediction. Among the 78 pairs, 16 drug pairs were confirmed to have synergistic effects, which are illustrated as red dots. The examination of the top 20 predictions detected five positive pairs through DIGRE, seven positive pairs through SynGen, DrugComboRanker and Zhao's method, and successfully predicted 11 positive pairs through RACS. More details are described in Supplementary Table 4.

The DREAM experiment also provides a solid reference by which to investigate the contributions of transcription profiles in the RACS model. The application of transcriptomic filters increased the PC-index for DREAM data from 0.69 to 0.78, with improvements in the AUC value from 0.783 to 0.853. Compared with random change, which showed a PC-index of 0.5 and an AUC value of 0.5, an 18% improvement in the PC-index and a 14% improvement in the AUC were achieved through the inclusion of transcriptomic filters. Interestingly, the positive pairs were maintained during the filtering process, whereas the non-synergistic pairs or true negatives according to the DREAM results were removed (indicated as triangles in Fig. 2b4). This result suggests that the incorporation of transcriptomic filters can notably improve the ranking results by greatly reducing the number of false positives.

### Significant ranking ability on breast and lung cancer cells

RACS was further evaluated using the ER-positive breast cancer cell line MCF7. A total of 118 anticancer agents (FDA-approved or under clinical trial) were reshuffled into binary pairs. After removing the 26 known synergistic pairs (Supplementary Table 5), 6,877 pairs remained as unlabelled data. To gain a more reliable ranking for experimental validation, RACS was constructed for 30 times using different numbers of positive pairs, and those that consistently appeared at the top 1% of the list were compiled into a consensus rank via Spearman's footrule distance to obtain the preliminary ranking list^16^ (Supplementary Fig. 1, Supplementary Table 6). After filtering based on the drug-perturbated transcription profile of MCF7 cells (Supplementary Table 7), 33 out of the 41 drug pairs were on the final ranking list (Supplementary Table 6). Pairs for which an expression profile was available for only one drug were still included in the list. A literature search indicated five combinations with synergistic anticancer effects, namely curcumin and resveratrol on colorectal cancer^17^, trastuzumab and erlotinib on breast cancer^18^, topotecan and vorinostat on small cell lung cancer^19^, estramustine and docetaxel on breast cancer^20^ and bleomycin and etoposide on endometrial carcinoma^21^ (Supplementary Table 6). The antibody agents and those not commercially available were removed from the list. The drugs in the remaining 17 pairs were purchased and experimentally validated using the human MCF7 cell line (Supplementary Note 1).

**Figure 3:**
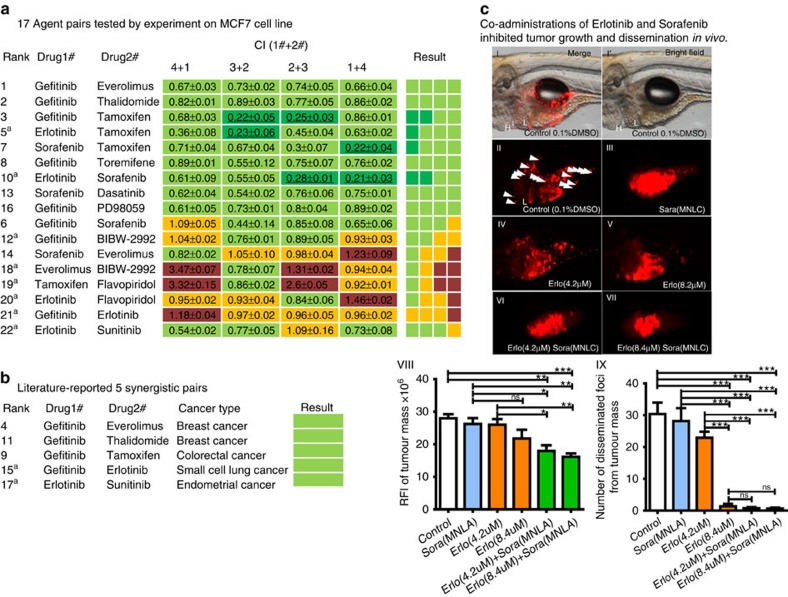
Validation of the prediction result on ER positive breast cancer cell line MCF7. (**a**) Seventeen agent pairs tested by experiment on MCF7 cell line. CI for each drug pair was summarized in a heat map. Green indicates synergy (CI<0.9); dark green indicates strong synergy (CI<0.3); yellow indicates additive (0.9<CI<1.1); and red indicates antagonism (CI>1.1). ‘4+1', ‘3+2', ‘2+3', and ‘1+4' indicate combinations of same drug pairs at four different concentration ratios: 4:1, 3:2, 2:3, and 1:4. The results summary table was rearranged according to the CI values in the order of ‘Strong synergy → synergy → Additive effect → Antagonism'. ^a^There involves single agent without MCF7 expression profile. (**b**) Five agent pairs reported in literatures. (**c**) Co-administrations of Erlotinib and Sorafenib inhibited tumour growth and dissemination in a zebrafish-based human cancer cell xenograft model. (I-VII) MCF-7 cells were labelled with red fluorescence for easy observation in the zebrafish tumour xenograft model. Compared with vehicle control treated embryos, Erlotinib combined with Sorafenib significantly inhibited tumour growth and dissemination in 4-dpi zebrafish embryos. White arrowheads indicate disseminated tumour foci. H, heart; L, liver. (VIII,IX) Quantification of tumour growth and numbers of disseminated tumour foci. Columns, mean; bars, s.e.m. (*n*=9; ANOVA). RFI, relative fluorescence intensity; dpi, days post injection. ns: not significant, *P value<0.05, **P value<0.01, ***P value<0.0001.

Two methods are commonly used to identify the expected dose–response relationship for combination therapy compared with monotherapy^22^. In the present study, the combination index (CI) according to Chou and Talalay^23^ was considered for measuring cooperative effects, but drug synergy was defined via stricter criteria. A pair was only accepted as synergistic when the CI values were all <0.9 for four combinational concentrations, instead of the usual CI cut off of 1 for one combination. Based on the stringent criteria, nine of the 17 pairs (52.94%) were newly identified as synergistic in inhibiting MCF7 cell proliferation (Fig. 3a, Supplementary Fig. 2). After addition of the five pairs from the literature (Fig. 3b), 63.64% (14/22) of the predicted pairs were confirmed to exert synergistic anticancer effects at the cell line level, and 57.89% (11/19) experimentally showed synergistic effects on MCF7 cells. Overall, the true synergistic combinations with literature support and cell-line experimental confirmation were all within the top 5‰ of the prediction results. Interestingly, four of the nine (44%) synergistic agents showed strong synergistic effects (with CI<0.3), and these molecules were ranked in the top 2‰. Notably, as shown in Fig. 3a, the predictions of pairs with expression profiles for both drugs are markedly better than that of those with expression profiles from only one drug, further indicating the importance of incorporating expression profile information.

As a control, 30 compound pairs were randomly selected from the unlabelled samples, and the same experimental protocol was applied. Only four of the 30 pairs (13.33%) showed synergistic effects (Supplementary Figs 3 and 4), and none of these compounds showed strong synergy. The pick-up rate (number of experimentally confirmed pairs/total number of the to-be-validated drug pairs) of RACS was significantly higher than that of the randomly selected pairs (*P*-value=0.006188; Fisher's exact test). RACS was also compared with one-class SVM^24^, which can solve similar problems. The comparison showed that RACS is a markedly better technique than one-class SVM as indicated by the true positive rate, hit-rate and enrichment factor (Supplementary Note 2). The substantial robustness of RACS was determined by testing on a simulated CN through the random deletion of nodes from the network (Supplementary Note 3). We examined the model performance of each individual feature among the seven selected ones on different positive samples (Supplementary Note 4). Other feature combinations were also explored (Supplementary Note 5, Supplementary Fig. 11). And the results showed that the combination of all seven features generated the best performance. In addition, reducing the number of features but increasing the number of positive samples decreased the performance of RACS (Supplementary Note 2). Moreover, the evaluation of RACS through the sub-selection of target proteins also revealed the robustness of this model (Supplementary Note 6 and Supplementary Fig. 12).

The network targeting modes of the synergistic drugs were reviewed in the context of gene expression and the mutational profile of the MCF7 cell line (Supplementary Fig. 13, Supplementary Note 7). All of the synergistic drugs simultaneously targeted molecules upstream of cell membrane receptors as well as important cancer cross-talk pathways in the cytoplasm. A total of seven out of nine pairs inhibited the Ras and oxidative stress pathways or the Ras and PI3K/Akt/mTOR signalling pathways in a complementary manner. Interestingly, the strong synergistic pairs simultaneously targeted more than two classes of membrane receptors. This result may reflect the drug frequency in the collected synergistic pairs. The most frequently observed drug in all nine synergistic pairs was gefitinib, a compound typically used to treat non-small-cell lung carcinoma through targeting epidermal growth factor receptor (EGFR). The second most frequently observed drug was tamoxifen, a drug commonly used to treat breast cancer, which targets the estrogen receptor in breast tissue (Fig. 3).

RACS was further validated using the human lung adenocarcinoma cell line A549. In this experiment, 11 drugs were analysed based on their gene expression profiles and target information (Supplementary Table 8). The same procedure and standards were used to rank the 55 potential drug pairs. The results in the top 10% and bottom 10% (six agent pairs) were experimentally evaluated (Supplementary Note 8). The results showed that two drug pairs (33.33%), namely gefitinib with quinacrine and erlotinib with quinacrine, showed strong synergistic effects (with CI<0.3) on the inhibition of A549 cell proliferation (Supplementary Fig. 14a, Supplementary Fig. 15). In contrast, none of the six pairs in the bottom of the ranking showed synergistic effects (Supplementary Figs 14b and 16). Surprisingly, the first two pairs that included rosiglitazone showed antagonistic effects. The cell line results obtained in the present study showed that A549 cell proliferation is non-sensitive to rosiglitazone treatment at low concentrations, whereas a high concentration of 40 μM only induced 20–30% inhibition of cell growth^25^. Thus, the half inhibitory concentration (IC_50_) concentration of rosiglitazone may not be detectable in this testing range, generating bias in the calculation of the synergy score (CI index) between rosiglitazone and other drugs, which is consistent with the results of previous studies^25^.

### Validation of drug synergy and potential toxicity *in vivo*

Because anticancer effects often involve cytotoxicity, further screening should be applied to identify strong synergistic combinations with low toxicity and few side effects on normal cells or organs. To further validate the obtained results *in vivo*, four compounds covering the four strong synergistic pairs shown in Fig. 3a, namely gefitinib, erlotinib, sorafenib and tamoxifen, were selected to examine their drug synergistic effects and potential toxicity using a zebrafish-based human cancer cell xenograft model (Supplementary Note 9)^26^,^27^. We observed that zebrafish are tolerant to individual treatment with gefitinib or erlotinib at the same concentration that was used in the cellular experiments, as indicated. However, tamoxifen and sorafenib showed severe toxicity in the zebrafish model (Supplementary Figs 17 and 18). The LC50 and maximum non-lethal concentration (MNLC) on zebrafish were measured as 1.37 and 0.96 μM, respectively, for sorafenib and 5.35 and 3.19 μM, respectively, for tamoxifen. The MNLC concentrations of sorafenib and tamoxifen were subsequently used in combination with other compounds in the zebrafish experiments.

As indicated in Fig. 3c, the combination of erlotinib and sorafenib showed a significant synergistic effect on the inhibition of xenografted MCF-7 cell proliferation in zebrafish, with no apparent side effects. In contrast, treatment with each individual compound showed no effects on tumour mass compared with the vehicle control. Notably, sorafenib alone reduced the dissemination of MCF-7 cells in zebrafish, indicating that this drug inhibits the metastasis of breast cancer, consistent with the results obtained in recently published studies^28^,^29^. Interestingly, the other three combinations containing tamoxifen showed severe toxicity to zebrafish and caused 100% lethality, although each compound alone at the experimental concentration showed no harmful effect on the fish (Supplementary Fig. 19). This finding also suggested that the tamoxifen combinations exerted synergetic cytotoxic effect on normal cells. Thus, the zebrafish-based *in vivo* study provides additional information for screening drug combinations with potentially severe side effects.

## Discussion

Few studies or established methods have achieved the successful prediction of drug synergy. According to DREAM data, the current best method performs only slightly better than random chance, indicating a need for improvement^8^. To save time and facilitate wet-lab screening, an effective model, denoted RACS, was established in this study to predict drug synergy in terms of anticancer effects. Extensive validation of the synergistic effects on three types of cancers showed that the proposed data-driven model, compared with the hypothesis-driven model, markedly increased the prediction power of drug synergy calculation. RACS is based on similarities to positive samples in the space of multiple features empirically derived from differentiating synergistic and non-synergistic drug combinations on cancer. The initiating features for the RACS model cover indices of molecular and pharmacological characteristics, drug-targeting networks, and gene expression profiles in drug responses. A statistical analysis showed that the molecular structure and pharmacology features of the drugs had no significant correlation with drug synergy in this dataset, in contrast with the results of a previous report^12^. However, the results of the present study are consistent with the findings from the DREAM analysis, showing that synergy is not a universal property derived from chemical, structural or substrate information^8^. Targeting modes on biological networks and genomic features of gene expression play important roles in determining drug synergy. Notably, the incorporation of gene expression profile information markedly reduced the number of false positive results, which may reflect the fact that the synergistic drug agents may need to alter gene expression in a correlated manner to produce synergistic effects.

Previous models were formulated based on different hypotheses. Some of these models, such as Zhao's method and DIGRE, primarily consider similarities between genomic changes on treatment with different drugs. In contrast, other methods consider additional information; for example, DrugComboRanker utilizes disease-specific signalling networks and the SynGen model considers cellular phenotyping data. A complementary concept was also highlighted in both DrugComboRanker and SynGen. We strongly agree that the drug mechanism of action is critical to drug synergy. Because drug targets play fundamental roles in the MOA, we described the MOA of drugs through mapping targets to the commonly accepted CN. Thus, the functional influence of combinational targeting modes under drug perturbation can theoretically be compared in a quantitative manner using the same network. Because genomic expression profiles are highly dynamic and context dependent, we can use this feature as a second filter to refine the ranked list. In general, the difference between RACS and other top-performing algorithms may lie in two aspects: the positive data set of synergistic combinations on different cancers, which facilitates the statistic derivation of a whole set of significant features for further model formulation and the incorporation of the drug targeting network to describe the MOA of drugs, which covers complementary and non-complementary mechanisms^3^,^7^,^11^,^12^.

In the present study, different evaluation parameters and methods were adopted according to different testing scenarios. Typically, the true positive rate and AUC value are adopted to evaluate the performance of prediction models. The true positive rate is usually calculated under a certain cut-off, whereas the AUC value represents the overall performance of a model under a varied cut-off. To evaluate the ranking model of drug synergy, the DREAM consortium proposed a new parameter, the PC-index, to avoid the noise of experimental replications. The analysis of 78 pairs using DREAM data revealed that the PC-index is much more rigorous than the AUC value as an overall indicator for evaluating model performance. The prominent advantage of the PC-index compared with the AUC value is its anti-resistance to data perturbation. For instance, the AUC value of the DIGER method decreased from 65 to 48% after removal of mitomycin C from the set of 14 drugs. However, the PC-index of DIGER and Zhao's method remained consistent or almost the same, indicating the stability of the PC-index to compound perturbation. However, the PC-index may not fit the results for breast or lung cancer, for which multiple combinations of different concentrations were used instead of biological replicates. As illustrated in Fig. 3, the biological effects of the same drug combination can either be synergistic, additive or even antagonistic on the same cell line at different concentrations. In addition, the AUC value is not suitable, because only partial drug pairs were experimentally examined. Thus, we simply adopted the true positive rate, consistent with previous studies. Indeed, because compound synergy is highly context-specific, robust evaluation metrics generalized for different experiment designs are still expected. Furthermore, DREAM data provide abundant information, including dose-response curves for cell viability, gene expression profiles (GEP) of baseline cells, and time-series GEP following each drug perturbation, together with the baseline genetic profile previously reported. Only this set of robust data allows the application of different methods and comprehensive comparisons between models. Thus, the performance of RACS on the analysis of DREAM data was compared with those of four different methods, namely SynGen, DIGRE, DrugComboRanker and Zhao's method. However, other public datasets often miss some information, preventing thorough comparisons. For example, the best method from DREAM (DIGRE) requires the gene expression profiles obtained after individual drug treatment, which is information that is lacking in the MCF7 and A549 datasets obtained in the present study.

A number of previous studies have discussed the synergistic mechanisms of drug combinations^11^. Because different datasets in different contexts may show different results, various molecular patterns underlying the targets of drugs on biological pathways or networks have been described. The synergistic drug pairs identified in the present study were found to modulate both pathways upstream of cell membrane receptors and downstream cross-talk cancer pathways in the cytosol, likely reflecting the fact that many of the available anticancer drugs are designed based on membrane receptors. Because intracellular pathways are typically regulated through signals upstream from membrane receptors, the inhibition of membrane receptors together with effects at intracellular sites may lead to strong inhibition of cell proliferation. In addition, strong synergistic pairs that targeting more types of membrane receptors may enhance the inhibition of the downstream Ras signalling pathway, which may further amplify the drug synergy.

Antagonism was also observed in the top ranked potentially synergistic drug pairs. The data for most of these drugs, such as BIBW-2992 and everolimus, included only partial information of the gene expression profiles after drug treatment. BIBW-2992 is an irreversible multi-target tyrosine kinase inhibitor designed directly for EGFR mutations. Because EGFR mutations have not been reported in MCF7 cells, BIBW-2992 may exert off-target effects in MCF7 cells, resulting in an antagonistic effect when combined with everolimus. Indeed, the combination of BIBW-2992 and everolimus, which is ranked No. 31 in Fig. 3, showed a typical context-dependent effect, in which the combinations of different concentrations induced different effects, including antagonism, addition, or synergism on the MCF7 cell line. The results shown in Supplementary Table 9 revealed that everolimus inhibits MCF7 cell proliferation in a dose-dependent manner, whereas BIBW-2992 does not. Everolimus inhibits the mTORC1 protein complex, resulting in the hyper-activation of AKT kinase via the mTORC1 negative-feedback loop^30^. In addition, BIBW 2992 may target various oncogenic signaling pathways in different cancer types, including PI3K/AKT. It has been reported that BIBW 2992 decreases *AKT* phosphorylation, even in afatinib-resistant cells^31^,^32^,^33^. However, an inhibition of *AKT* phosphorylation was observed with a sensitizing concentration in different cell lines^34^. We suspected that concentration sensitization may contribute to the different effects observed for the combination of BIBW-2992 and everolimus, but the detailed mechanism needs further validation. It is not certain whether these effects will be predicted through the gene expression signatures of the drugs, because the expression profile of BIBW-2992 in MCF7 cells was not obtained. This finding indicates that mutational and concentration-dependent data could also be incorporated to increase the performance of the computational model. Drug targeting patterns in the context of disease-specific pathways, the gene expression profiles induced by drug treatment, the drug-dose responses and genome mutation information may improve drug synergy predictions and should be incorporated for future improvements.

Synergistic mechanisms, such as pharmacokinetic potentiation with absorption, distribution, metabolism and excretion (ADME), coalistic mechanisms and pharcodynamically synergistic mechanisms, have been surveyed in several studies^11^. Notably, however, the RACS approach described in this manuscript addressed only the issue of pharmacodynamic synergy with protein/gene drug targets and DEGs from expression profiles of individual drugs in the same cell line. Pharmacokinetic and coalistic synergy models are currently not applicable in RACS, but both models should be amenable to future improvements.

In summary, in the present study, we proposed a set of features associated with drug synergy in cancer and formulated an efficient model, denoted RACS, for the prediction of synergistic drug combinations for cancer treatment. Through validation of the predicted interactions on three types of cancer cell lines and a fish model, we showed that RACS displays a high capacity to rank potential combinations that exert synergistic anticancer effects. As new discoveries of individual drugs become increasingly difficult, combinations of available drugs provide critical opportunities for treating cancer. With the increasing accumulation of patient data from TCGA and patient-derived xenograft (PDX) models, the workflow from RACS prediction to cell line validation and further *in vivo* model screening may accelerate the identification of personal combinational therapy through a reduction of the search space when repurposing existing drugs.

## Methods

### Datasets

A total of 41 known synergistic anticancer agents pairs that have entered clinical trials were initially obtained from the Drug Combination Database (DCDB)^10^ and the literatures in PubMed (Supplementary Table 5). Because some drug targets of the pairs cannot be successfully mapped to Kyoto Encyclopedia of Genes and Genomes (KEGG) pathways, these drugs pairs were removed from the initial list. In total, 26 pairs were considered as the final positive/labelled samples for further modelling in this study.

A total of 14 individual drugs/compounds were provided by the NCI-DREAM consortium^8^. As protein targets were observed for only 13 of the 14 agents, 78 pairwise combinations derived from these 13 agents were used as the testing dataset for the human β-cell lymphoma cell line OCI-LY3.

A total of 142 FDA-approved anticancer agents and those under clinical trials were initially retrieved from DrugBank version 3.0 (ref. 35), TTD (ref. 36), and PubMed (Supplementary Data 1). The key words include ‘*antineoplastic*', ‘*anticancer*', ‘*antitumor*', ‘*oncology drug*', ‘*cancer drug*', ‘*anticarcinoma*', ‘*anticarcinogen*', ‘*cancer fighting*' and derivative words. Subsequently, 118 agents remained after removing agents without gene ontology (GO) annotations or KEGG pathway information for the targets. Reshuffling of the 118 drugs produced 6,877 unlabelled pairs as the testing data for the lung adenocarcinoma cell line A549 and the ER-positive breast cancer cell line MCF7. The target proteins for all of the drugs were retrieved from DrugBank version 3.0, TTD, and PubMed.

### Features of the preliminary ranking model

We initially prepared a candidate list of 14 features to describe the synergistic characteristics of drug pairs in our prediction model. These 14 features were designed to cover the comprehensive molecular and pharmacological characteristics of each drug as well as their target pathways in a systematic manner. A *Z*-score test was then performed to select those features that significantly differed between the synergistic drug pairs and the unlabelled combinations (*|Z*-score|>3, All were normalized to [0,1]), generating the seven features listed below:

(1) GO-based Mutual Information Entropy (MI): This feature indicates the similarity between the biological processes (BPs) regulated by the targets of the two agents. Larger values often imply higher functional similarity. The features were calculated as follows: First, 1,006 GO terms of BPs were retrieved as the cancer-related BPs for genes in the KEGG ‘*Pathways in cancer*' using the online tool DAVID^37^ with *P* value<0.05; Modified Fisher's exact test (Supplementary Data 2). Second, the target proteins of each agent were mapped to cancer-related BPs to form a 1,006-dimensional binarized fingerprint, with the element *‘1'* indicating an existing of drug target. Third, the relationships between the cancer-related BP fingerprints of paired agents were calculated using mutual information entropy. Here the GO-based mutual information entropy for agent *x* and agent *y* was defined as


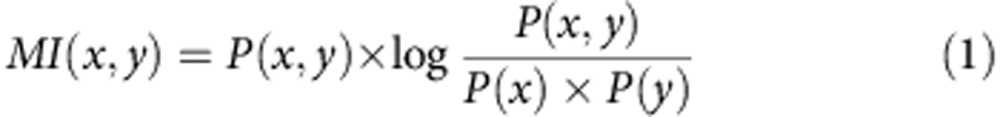


where *P*(*x*) is the ratio of mapped GO terms in cancer-related BPs for agent *x, P*(*y*) is the ratio of mapped GO terms in cancer-related BPs for agent *y*, and *P*(*x,y*) is the ratio of the common GO terms mapped to cancer-related BPs between *x* and *y*.

(2) Distance (Dis): This feature represents the average distance between the target proteins of the two agents in the context of a protein–protein interaction (PPI) network. This distance was defined as the average distance between two groups of target proteins:


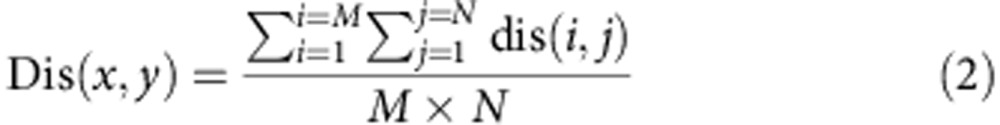


where dis(*i, j*) is the shortest path between the *i*th target protein of agent *x* and the *j*th target protein of agent *y* in the background PPI network (Supplementary Note 6). *M* and *N* represent the numbers of target proteins of agents *x* and *y*, respectively.

(3) Drug Combination Interference (DCI): This feature represents the variance between the combined agent pairs and the sum of the individual agents in the effect on network information-transmitting efficiency. According to the DCI, the synergistic combinations tend to produce more effects on the information-sending efficacy of the CN than the sum of the individual drugs. DCI was calculated based on the relative efficiency change (Δ*E*) of the CN (Supplementary Note 10) in sending information after combined drug treatment^38^:


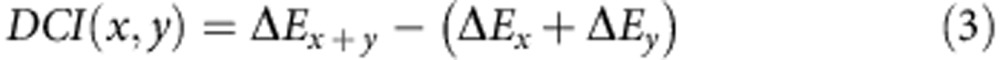


Δ*E* for agent *x* is calculated as:


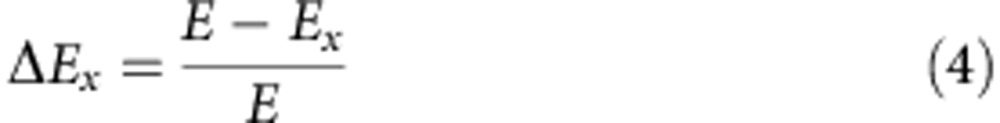


*E* represents the information-sending efficacy of the CN without agent perturbation and was calculated as the arithmetic mean of all of the shortest distances between each pair of nodes in the CN. *E*_*x*_ was calculated as the information-sending efficacy of the network derived after removal of all the targets of agent *x* from the original CN.

(4) Efficacy (Eff.D, Eff.B and Eff.E): These features represent the efficacy of drug pairs considering their therapeutic effects as well as additional effects and were calculated based on the degree/betweenness/eigenvector centrality of the drug target in the network. These three features were designed based on the assumption that good combinations generate maximum therapeutic effects and minimum additional effects. The efficacy (Eff) was designed for hitting critical targets in the CN while avoiding hitting targets in non-cancer networks (NCNs):


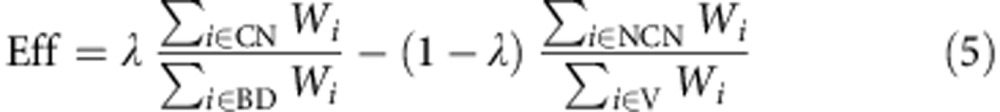


The first part of the formula represents therapeutic effects, whereas the second part of the formula represents additional effects. *λ*∈[0,1] is used to balance the two parts of the formula. Here *λ* is set to 0.1, which is the value that can best distinguish the known anticancer synergistic agents from the unlabelled/background samples. Different weights of node *i* (*W*_i_) were calculated as the degree, betweenness, and eigenvector centrality of the target node in the network to form the three different Effs (Eff.D, Eff.B, Eff.E).

CN: target proteins in the cancer network;

BD: target proteins in the background PPI network including the CN;

NCN: target proteins within BD but outside of the CN;

*V*: all nodes in the background PPI network including the CN.

(5) Unrelated mapped pathway pairs (MP.U): Unrelated pathways pairs are defined as pathway pairs that are not identical, not cross-talking, and not interacting (please refer to Supplementary Note 11 for the definitions of identical, cross-talking and interacting pathway pairs) according to KEGG pathway information. For an agent pair, MP.U was calculated as the proportion of unrelated pathway pairs among the total number of pathway pairs targeted by each drug.

### Preliminary ranking system

A well-defined semi-supervised learning method incorporating a manifold ranking algorithm^14^ was applied as a preliminary ranking system according to similarities with positive samples in the seven-feature space (Supplementary Note 12). All of the positive and unlabelled samples are represented based on the aforementioned seven features to formulate the model. The performance of manifold ranking was assessed using different numbers and compositions of positive samples as bait to build the model (Supplementary Note 2). Detailed information on the manifold ranking can be accessed in Supplementary Materials.

### Secondary filtering system

Two parameters derived from gene expression profiles were identified to further filter the ranked results obtained from the primary ranking system. (*P* value<0.05, Permutation Test; Supplementary Note 13):

DEG_Overlap was calculated as:


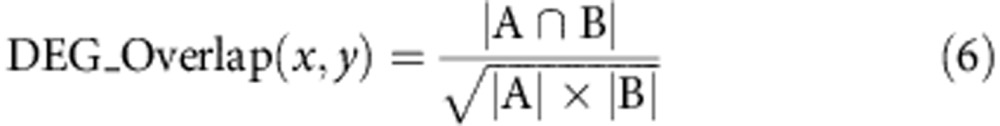


where **A** and **B** represent the DEG sets perturbed by agent *x* and *y*, respectively.

Pathway_Coverage was defined as:


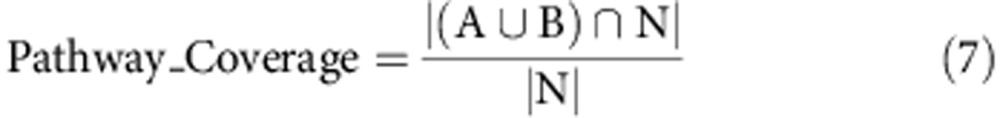


where **A** and **B** represent the DEG genes upon treatment with agents *x* and *y*, respectively. **N** denotes all of the genes covered by the specific cancer pathway (Supplementary Note 7).

These two parameters were calculated for each drug pair. The drug pairs with *P* values<0.05, Permutation Test; for both of these parameters were retained after filtering (please refer to Supplementary Note 13 for the *P* value calculation).

### Validation of drug synergy *in vitro*

Human breast cancer cell line MCF7 was obtained from American Tissue Type Culture Collection (ATCC, Rockville, MD) and maintained in a humidified 37 °C atmosphere containing 5% CO2 and cultured in DMEM supplemented with 10% fetal bovine serum (FBS). NSCLC cell lines A549 was obtained from the Shanghai Cell Bank, Chinese Academy of Sciences and grown in F12K mediums supplemented with 10% FBS and 100 μg ml^−1^ penicillin and 100 μg ml^−1^ streptomycin at 37 °C under 5% CO_2_. Dasatinib, erlotinib, everolimus, gefitinib, sorafenib, sunitinib, azacitidine, cycloleucine, cytarabine, decitabine, etoposide, genistein, ifosfamide, melatonin, parthenolide, resveratrol, tamibarotene, terazosin, tretinoin, quinacrine, rosiglitazoneand tamoxifen were purchased from Biovision (Mountain View, CA). BIBW-2992, flavopiridol, PD98059, thalidomide, lapatinib, dexamethasone, imatinib, and toremifene were purchased from Selleckchem (Houston, TX, USA). The purity of each drug is above 98%. Drugs were dissolved in cell culture medium. The drug was used alone or in combination with the other drug at 4 different concentration ratios: 4:1, 3:2, 2:3, and 1:4. The cytotoxicity of each drug or combination was evaluated by MTT assay. To calculate the IC50, the single drug or the combined drug pair at the four different concentration ratios were diluted 1:4 with cell culture medium into six concentration gradients. For each concentration gradient, there were three replicates, and each experiment was repeated three times. The CI was introduced to determine whether a pair of drug combinations could produce synergy. Generally, it is considered that a CI value <0.9 indicates synergism, 0.9<CI<1.1 indicates an additive effect, and CI>1.1 indicates antagonism. Here an agent pair was recognized as synergistic if all of the four CI values calculated from the four different concentration ratios were <0.9.

### Validation of drug synergy and potential toxicity *in vivo*

Cell Tracker CM-DiI was purchased from Invitrogen. Zebrafish were raised and maintained under standard conditions^39^. Embryos were staged according to the work of Kimmel *et al*.^40^ The establishment and characterization of the fli1a-EGFP; Casper transgenic lines has been described elsewhere^41^,^42^. The zebrafish facility at Shanghai Research Center for Model Organisms is accredited by the Association for Assessment and Accreditation of Laboratory Animal Care (AAALAC) International. All animal experiments were approved by the Institutional Animal Care and Use Committee of Shanghai Research Center for Model Organisms (IACUC NO.2015-0012).

To determine the Maximum non-lethal concentration (MNLC) and LC_50_ of lead compounds, zebrafish were treated from 2-dpf to 5-dpf and mortality was recorded every 24 h. Dead zebrafish was defined as the absence of heartbeat under a dissecting stereomicroscope (Nikon SMZ645; Japan). Mortality curves were generated using GraphPad Prism 5.0 (GraphPad Software, San Diego, CA, USA) and the MNLC was determined with logistic regression. Zebrafish embryos were obtained using standard mating conditions and staged for cell xenotransplantation at 48 h post fertilization. Cancer cells were stained with 5 μg ml^−1^ CM-DiI diluted in PBS and washed four times and kept on ice before injection. Embryos were dechorionized using micro-forceps and anaesthetized with 0.0016% tricaine and positioned on their right side on a wet 1.0% agarose pad. Approximately 200 cancer cells were injected into the yolk sac. Injected embryos were transferred to a six-well plate (BD Falcon) containing drug of interest diluted in 5-ml fresh fish water and maintained at 31 °C for up to 4 dpi. Embryos and larvae were analyzed with Nikon SMZ 1500 Fluorescence microscope and subsequently photographed with digital cameras. Quantitative image analyses were performed using image based morphometric analysis (NIS-Elements D3.1, Japan). A subset of images was adjusted for levels, brightness, contrast, hue and saturation with Adobe Photoshop 7.0 software (Adobe, San Jose, California) to optimally visualize the expression patterns.

All data are presented as mean±s.e.m. Statistical analysis and graphical representation of the data were performed using GraphPad Prism 5.0 (GraphPad Software, San Diego, CA). Statistical significance was performed using a ANOVA or *χ*^2^-test as appropriate. Statistical significance is indicated by *P<0.05, **P<0.01 and***P<0.0001.

### Code availability

The program code is available at GitHub (https://github.com/DrugCombination/RACS).

## Additional information

**How to cite this article:** Sun, Y. *et al*. Combining genomic and network characteristics for extended capability in predicting synergistic drugs for cancer. *Nat. Commun*. 6:8481 doi: 10.1038/ncomms9481 (2015).

## Supplementary Material

Supplementary Information Supplementary Figures 1-21, Supplementary Tables 1-9, Supplementary Notes 1-13 and Supplementary References

Supplementary Data 1 118 agents forming into testing data for RACS. 118 FDA-approved anticancer agents and those under clinical trials were retrieved from DrugBank version 3.0, TTD, and PubMed. Target proteins for all the drugs were also retrieved from DrugBank version 3.0, TTD, and PubMed. Reshuffling of the 118 drugs produced 6,877 unlabeled pairs for further studies on lung adenocarcinoma cell line A549 and ER-positive breast cancer cell line MCF7.

Supplementary Data 2 Cancer-related BPs. 1,006 GO terms of BPs (biological processes) were retrieved as the cancer-related BPs for genes in the KEGG 'Pathways in cancer' using the online tool DAVID.

Supplementary Data 3 Cancer-related pathways. 132 pathways were obtained as cancer-related pathways for genes in the KEGG 'Pathways in cancer' using online tool DAVID.

## Figures and Tables

**Table 1:** Network features formulating preliminary ranking system in RACS.

No.	Newly designed	Feature	Mean (Labelled samples)	Mean (Unlabelled samples)	Z-score (labelled samples versus unlabelled samples)	Indication
**1**	×	*Dis* (Distance)	2.48	2.59	−4.68	The average distance between the two groups of target proteins for the paired agents in the context of PPI network.
**2**	✓	*MP.U* (Unrelated mapped pathways)	1.23E–3	4.32E–4	3.22	The proportion of unrelated pathways regulated by the targets of the two agents.
**3**	✓	*MI* (GO-based Mutual Information Entropy)	−1.22E–3	4.75E–4	−4.04	The similarity between the biological processes (BPs) regulated by the targets of the two agents.
**4**	✓	*Eff.D* (Efficacy)	0.23	0.16	5.61	The evaluation of the efficacy of drug pairs considering both therapeutic effects and additional effects, calculated with degree of the drug targets in the network.
**5**	✓	*Eff.B* (Efficacy)	0.17	0.12	4.34	The evaluation of the efficacy of drug pairs considering both therapeutic effects and additional effects, calculated with betweeness of the drug targets in the network.
**6**	✓	*Eff.E* (Efficacy)	0.23	0.19	3.12	The evaluation of the efficacy of drug pairs considering both therapeutic effects and additional effects, calculated with eigenvector centrality of the drug targets in the network.
**7**	✓	*DCI* (Drug Combination Interference)	1.33E–3	–7.97E–4	3.77	The change of network information-transmitting efficiency between the combined treatment and the sum of individual agent treatment.

The *Z*-score test was applied to check the statistical significance between the synergistic drug pairs and the unlabelled combinations (with *|Z*-score|>3 as significant).
