# Supplementary material for: Combining genomic and network characteristics for extended capability in predicting synergistic drugs for cancer
Source: Nat Commun. 2015 Sep 28;6:8481. doi: 10.1038/ncomms9481 (PMC4598846; doi:10.1038/ncomms9481)
Supplement: Supplementary Information — Supplementary Figures 1-21, Supplementary Tables 1-9, Supplementary Notes 1-13 and Supplementary References [file ncomms9481-s1.pdf]

Supplementary information

Supplementary Figures

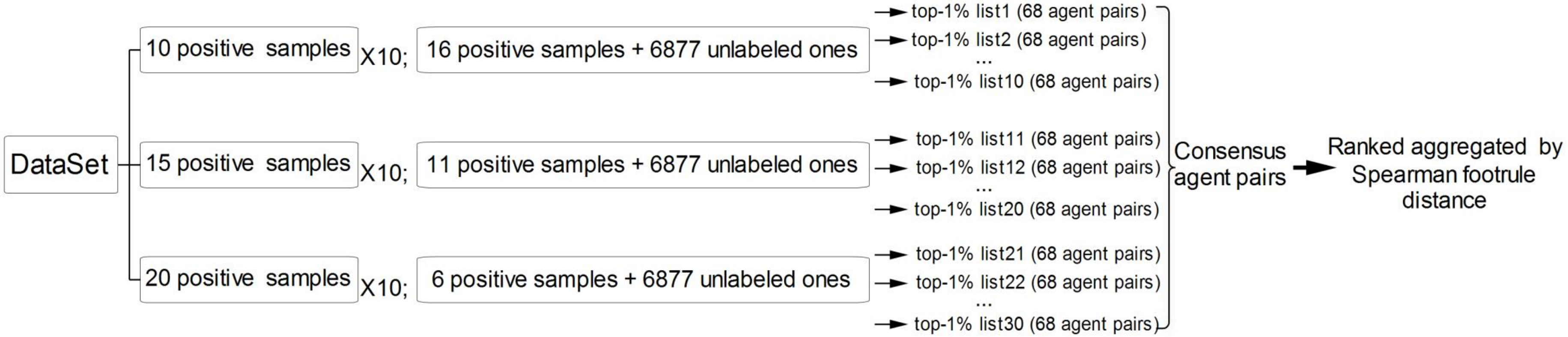

Supplementary Figure. 1: Construction of different "training" and "testing" datasets. There are no overlapped drug pairs between each set of "training" and the corresponding "testing" dataset.

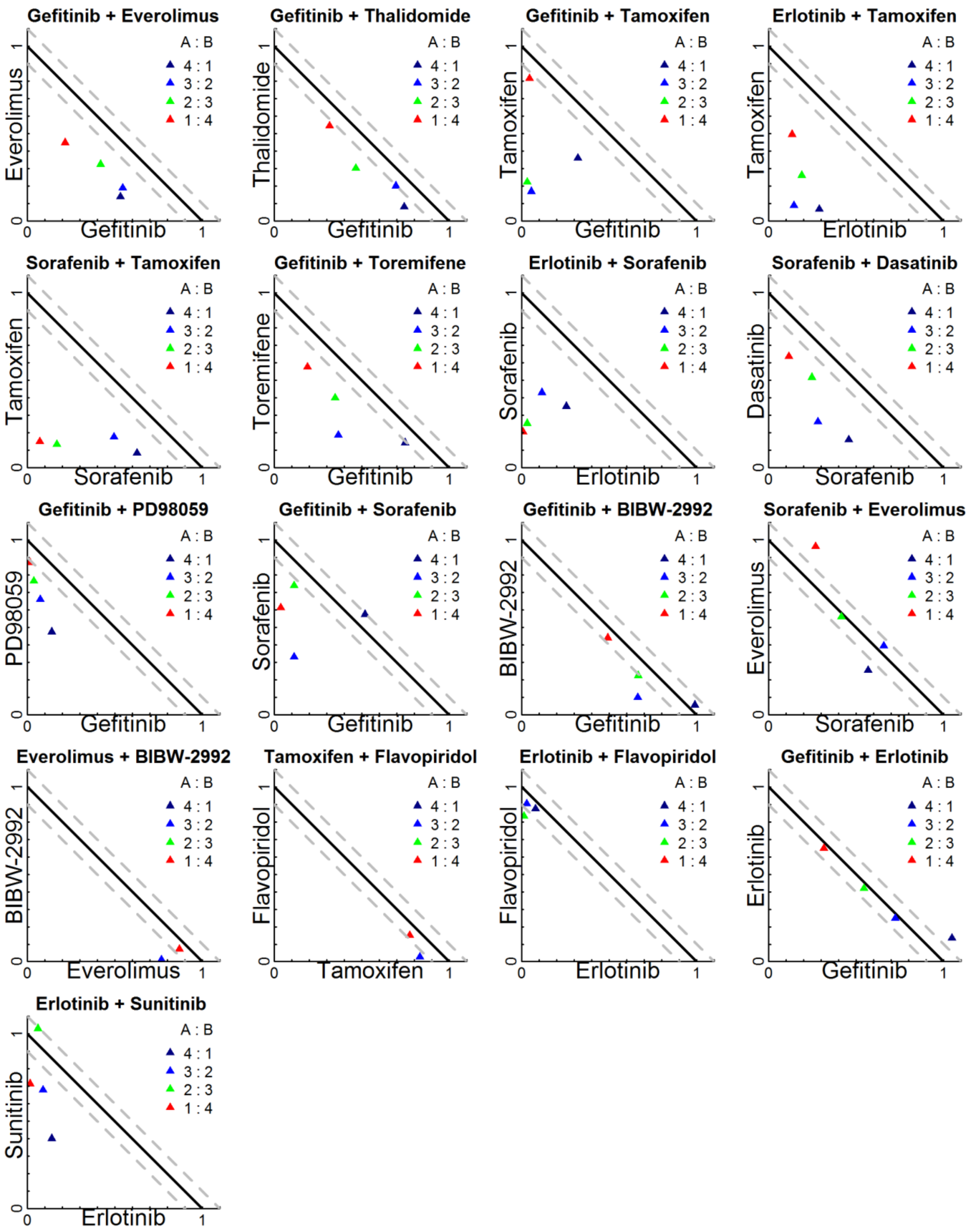

Supplementary Figure. 2: Isobolograms for the 17 agent pairs tested by experiment on MCF7 cell line. The horizontal axis and the vertical axis represent the relative dose of the drugs. The area between the two diagonal dotted gray lines in each plot represents the additive effect, while the triangular region under this additive effect area indicates the synergy effect. Triangular points represent the relative concentrations of the combinations resulting in 50% inhibition of cancer cells. The two drugs were used alone or in combinations at 4 different concentration ratios: 4:1, 3:2, 2:3, and 1:4. Therefore, there were four Triangular points for each drug pair.

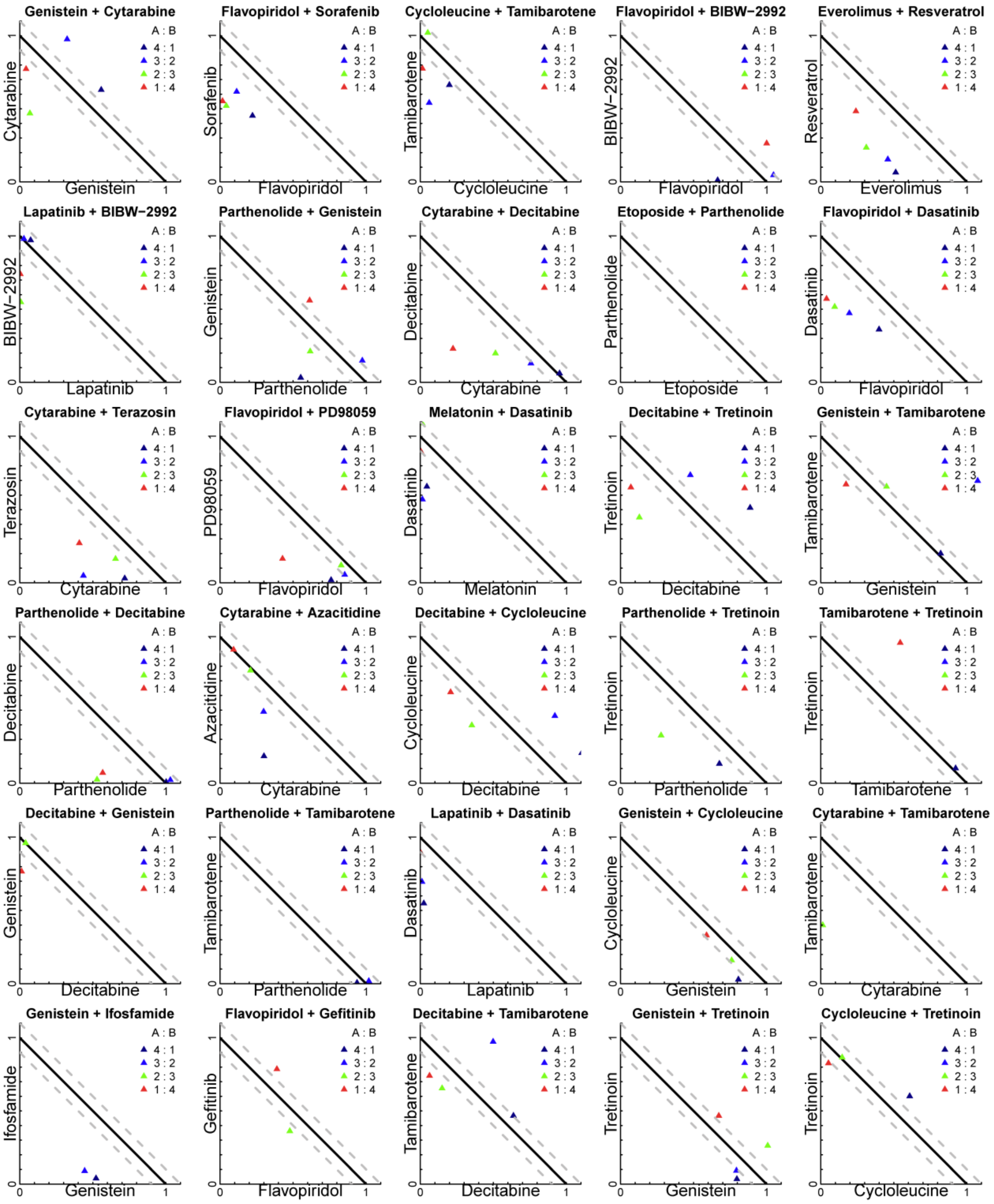

Supplementary Figure. 3: Isobolograms for the 30 randomly picked agent pairs tested by experiment on MCF7 cell line. The horizontal axis and the vertical axis represent the relative dose of the drugs. The area between the two diagonal dotted gray lines in each plot represents the additive effect, while the triangular region under this additive effect area indicates the synergy effect. Triangular points represent the relative concentrations of the combinations resulting in 50% inhibition of cancer cells. The two drugs were used alone or in combinations at 4 different concentration ratios: 4:1, 3:2, 2:3, and 1:4. Therefore, there were four Triangular points for each drug pair.

No.	Drug 1#	Drug 2#	CI (1# + 2#)				Result
			4+1	3+2	2+3	1+4	
1	Genistein (20μM)	Cytarabine (20μM)	1.19 ±0.17	1.30± 0.09	0.54± 0.05	0.82± 0.02	
2	Flavopiridol (4800nM)	Sorafenib (30μM)	0.67 ± 0.08	0.73 ± 0.24	0.56 ± 0.02	0.57 ± 0.11	
3	Cycloleucine (20μM)	Tamibarotene (20μM)	0.86± 0.14	0.60 ± 0.01	1.07 ± 0.07	0.79 ± 0.06	
4	Flavopiridol (4800nM)	BIBW-2992 (30nM)	0.68 ± 0.04	1.09 ± 0.04	1.51 ± 0.11	1.26 ± 0.06	
5	Everolimus (20μM)	Resveratrol (20μM)	0.58 ± 0.02	0.61 ± 0.05	0.55 ± 0.06	0.72 ± 0.12	
6	Lapatinib (20μM)	BIBW-2992 (30nM)	1.05 ± 0.04	1.01 ± 0.07	0.56 ± 0.08	0.74 ± 0.09	
7	Parthenolide (20μM)	Genistein (20μM)	0.58 ± 0.03	1.12 ± 0.11	0.83 ± 0.09	1.17 ± 0.09	
8	Cytarabine (20μM)	Decitabine (30μM)	1.01 ± 0.02	0.89 ± 0.13	0.71 ± 0.02	0.45 ± 0.03	
9	Etoposide (20μM)	Parthenolide (20μM)	6.58 ± 0.05	1.57 ± 0.18	1.42 ± 0.08	1.69 ± 0.23	
10	Flavopiridol (4800nM)	Dasatinib (840μM)	0.76 ±0.12	0.67 ±0.04	0.61 ±0.07	0.61 ±0.21	
11	Cytarabine (20μM)	Terazosin (20μM)	0.75 ±0.08	0.48 ±0.18	0.82 ±0.03	0.68 ±0.04	
12	Flavopiridol (4800nM)	PD98059 (240μM)	0.78 ±0.12	0.91 ±0.03	0.95 ±0.02	0.59 ±0.09	
13	Melatonin (20μM)	Dasatinib (840μM)	0.70 ±0.06	0.59 ±0.09	1.10 ±0.06	0.90 ±0.18	
14	Decitabine (30μM)	Tretinoin (20μM)	1.40 ±0.07	1.22 ±0.08	0.58 ±0.12	0.72 ±0.15	
15	Genistein (20μM)	Tamibarotene (20μM)	1.02 ±0.02	1.77 ±0.08	1.11 ±0.13	0.85 ±0.11	
16	Parthenolide (20μM)	Decitabine (30μM)	1.01 ±0.11	1.05 ±0.14	0.55 ±0.06	0.64 ±0.08	
17	Cytarabine (20μM)	Azacitidine (20μM)	0.49 ±0.17	0.79 ±0.22	0.98 ±0.03	1.01 ±0.02	
18	Decitabine (30μM)	Cycloleucine (20μM)	1.31 ±0.09	1.38 ±0.03	0.75 ±0.12	0.83 ±0.08	
19	Parthenolide (20μM)	Tretinoin (20μM)	0.81 ±0.07	2.32 ±0.01	0.61 ±0.07	7.21 ±0.16	
20	Tamibarotene (20μM)	Tretinoin (20μM)	1.03 ±0.14	2.03 ±0.10	2.29 ±0.29	1.50 ±0.05	
21	Decitabine (30μM)	Genistein (20μM)	1.51 ±0.09	1.57 ±0.05	1.00 ±0.02	0.78 ±0.21	
22	Parthenolide (20μM)	Tamibarotene (20μM)	0.94 ±0.18	1.04 ±0.33	1.86 ±0.08	1.48 ±0.05	
23	Lapatinib (20μM)	Dasatinib (840μM)	0.54 ±0.07	0.71 ±0.10	2.63 ±0.04	0.90 ±0.09	
24	Genistein (20μM)	Cycloleucine (20μM)	0.84 ±0.27	1.39 ±0.23	0.92 ±0.16	0.92 ±0.18	
25	Cytarabine (20μM)	Tamibarotene (20μM)	4.71 ±0.21	6.82 ±0.46	0.41 ±0.36	3.92 ±0.02	
26	Genistein (20μM)	Ifosfamide (20μM)	0.56 ±0.20	0.53 ±0.13	5.48 ±0.26	3.96 ±0.08	
27	Flavopiridol (4800nM)	Gefitinib (42μM)	1.38 ±0.10	1.59 ±0.06	0.84 ±0.21	1.18 ±0.01	
28	Decitabine (30μM)	Tamibarotene (20μM)	1.10 ±0.07	1.47 ±0.03	0.80 ±0.05	0.80 ±0.02	
29	Genistein (20μM)	Tretinoin (20μM)	0.83 ±0.23	0.89 ±0.16	1.27 ±0.06	1.14 ±0.16	
30	Cycloleucine (20μM)	Tretinoin (20μM)	1.21 ±0.06	2.22 ±0.15	1.01 ±0.10	0.88 ±0.12	

Supplementary Figure. 4: Result of the validation experiment for the randomly picked agent pairs on ER positive breast cancer cell line MCF7. Each drug with the corresponding maximum concentration used for the single drug in the experiment was recorded in **Drug1#** and **Drug2#**. The Chou and Talalay median-effect method was used to calculate the *CI* and summarized in a heat map. Green indicates synergy (CI < 0.9); yellow indicates additive (0.9 <CI <1.1); and red indicates antagonism (CI > 1.1). "4+1", "3+2", "2+3", and "1+4" indicate the two drugs were used in combinations at four different concentration ratios: 4:1, 3:2, 2:3, and 1:4.

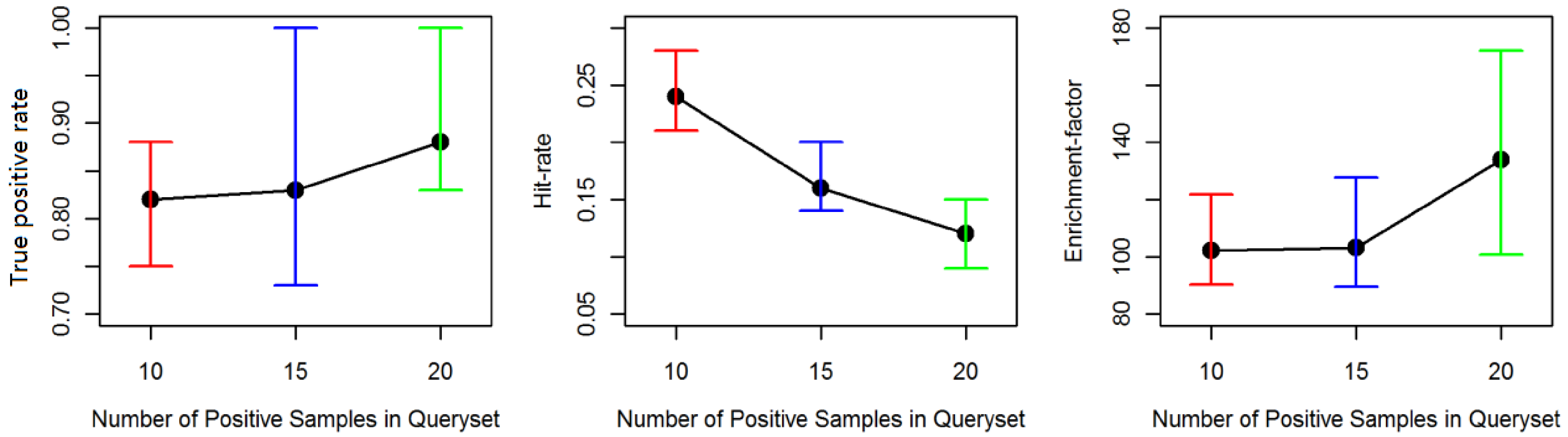

Supplementary Figure. 5: True positive rate, hit-rate, enrichment-factor of the preliminary ranking model of RACS. 10 times cross validation was performed with 10, 15, or 20 positive samples as training sets respectively. The dark dot is the mean value for true positive rate, hit-rate, and enrichment-factor, while the upper bound and lower bound denote the max and min value of them.

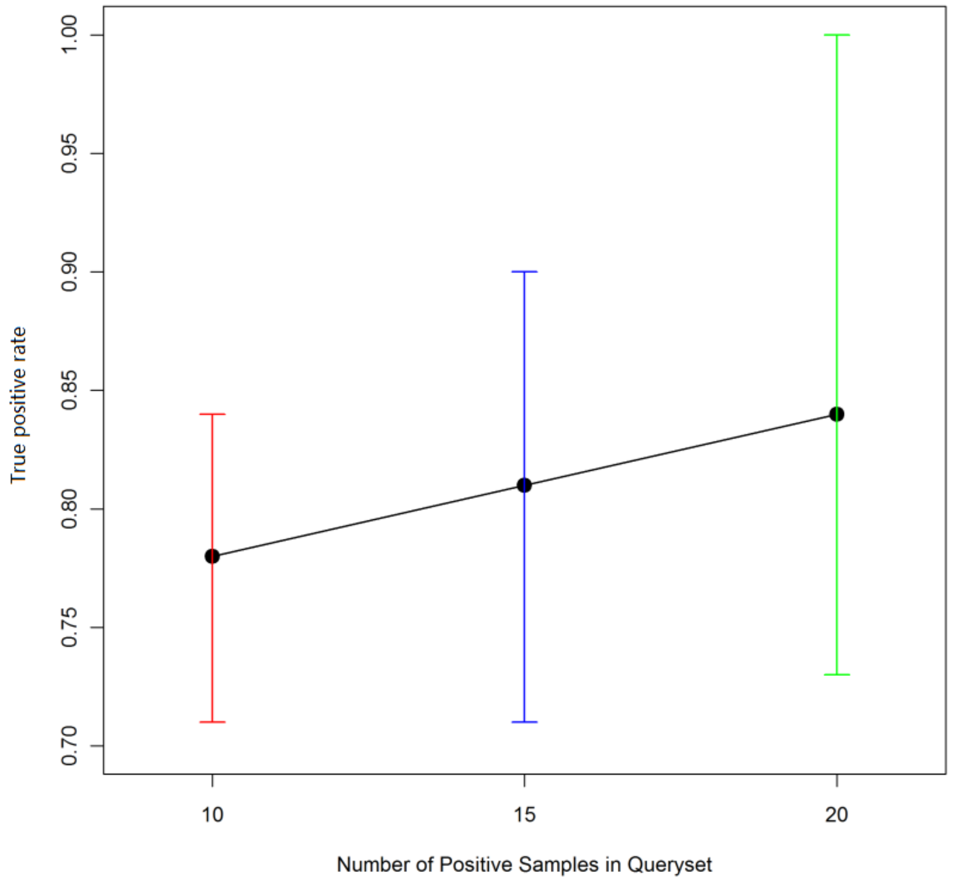

Supplementary Figure. 6: True positive rate for dataset with 41 positive samples and 5 features. 10 times cross validation was performed with 10, 20, or 30 positive samples as training sets respectively. The dark dot, the upper bound and lower bound represent the mean value, max and min value for true positive rate.

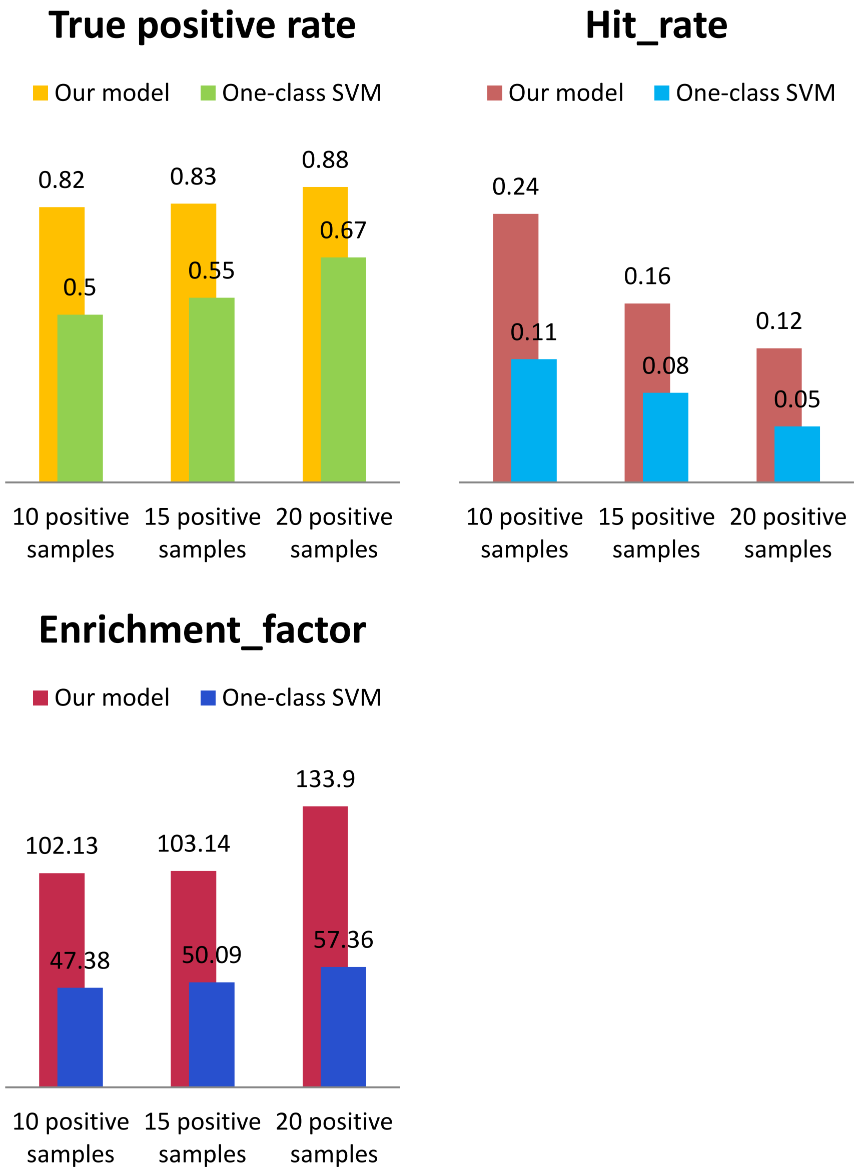

Supplementary Figure. 7: Comparison between RACS and one-class SVM. Values are averaged for 10 times.

Removed nodes	Mean (Dis)			Mean (Eff.D)			Mean (Eff.B)			Mean (Eff.E)			Mean (DCI)		
	Run-1	Run-2	Run-3	Run-1	Run-2	Run-3	Run-1	Run-2	Run-3	Run-1	Run-2	Run-3	Run-1	Run-2	Run-3
**0%**	2.48	2.48	2.48	0.23	0.23	0.23	0.17	0.17	0.17	0.23	0.23	0.23	1.33E-03	1.33E-03	1.33E-03
**1%**	2.5	2.53	2.51	0.22	0.21	0.22	0.16	0.16	0.16	0.23	0.23	0.23	2.01E-03	2.01E-03	2.11E-03
**5%**	2.51	2.54	2.52	0.21	0.2	0.2	0.16	0.16	0.16	0.22	0.22	0.22	2.13E-03	2.13E-03	2.16E-03
**10%**	2.52	2.56	2.54	0.2	0.19	0.2	0.15	0.15	0.15	0.2	0.2	0.2	3.40E-03	3.40E-03	3.50E-03
**20%**	2.53	2.59	2.57	0.2	0.18	0.19	0.15	0.15	0.15	0.2	0.18	0.19	5.59E-03	5.59E-03	5.59E-03
**30%**	2.58	2.63	2.6	0.19	0.18	0.18	0.14	0.14	0.14	0.19	0.18	0.19	3.84E-04	3.84E-04	4.08E-04
**40%**	2.58	2.65	2.63	0.19	0.18	0.18	0.13	0.13	0.13	0.19	0.18	0.18	8.42E-04	8.42E-04	7.84E-04
**50%**	2.59	2.68	2.65	0.18	0.17	0.18	0.12	0.11	0.11	0.18	0.17	0.18	3.86E-03	3.86E-03	3.96E-03
**60%**	2.64	2.73	2.69	0.17	0.17	0.17	0.11	0.1	0.1	0.17	0.17	0.17	-1.32E-03	-1.32E-03	-1.52E-03
**70%**	2.66	2.85	2.76	0.17	0.15	0.16	0.08	0.08	0.08	0.17	0.15	0.16	-3.88E-02	-3.88E-02	-4.20E-02
**80%**	2.74	2.93	2.81	0.17	0.15	0.16	0.07	0.07	0.07	0.17	0.15	0.16	-3.94E-02	-3.94E-02	-3.38E-02
**90%**	2.75	3.02	2.89	0.16	0.14	0.15	0.07	0.06	0.07	0.16	0.14	0.15	-1.54E-01	-1.54E-01	-1.35E-01

Z-score

- ∞ -4 -3 -2 -1 1 2 3 4 + ∞

Supplementary Figure. 8: The significance of *Dis*, *Eff.D*, *Eff.B*, *Eff.E*, and *DCI* based on the permutated *background network* and *cancer network*. Mean: the average of the various features for the 26 positive samples. Different percentages of nodes were randomly removed from the networks for 3 times. The significance of *Dis*, *Eff.D*, *Eff.B*, *Eff.E* was examined based on the permutated *background network*, while the significance of *DCI* was examined based on the permutated *cancer network*. Z-Score was used to assess the significance of the features and summarized in a heatmap (with |Z-Score|>3 as statistically significant).

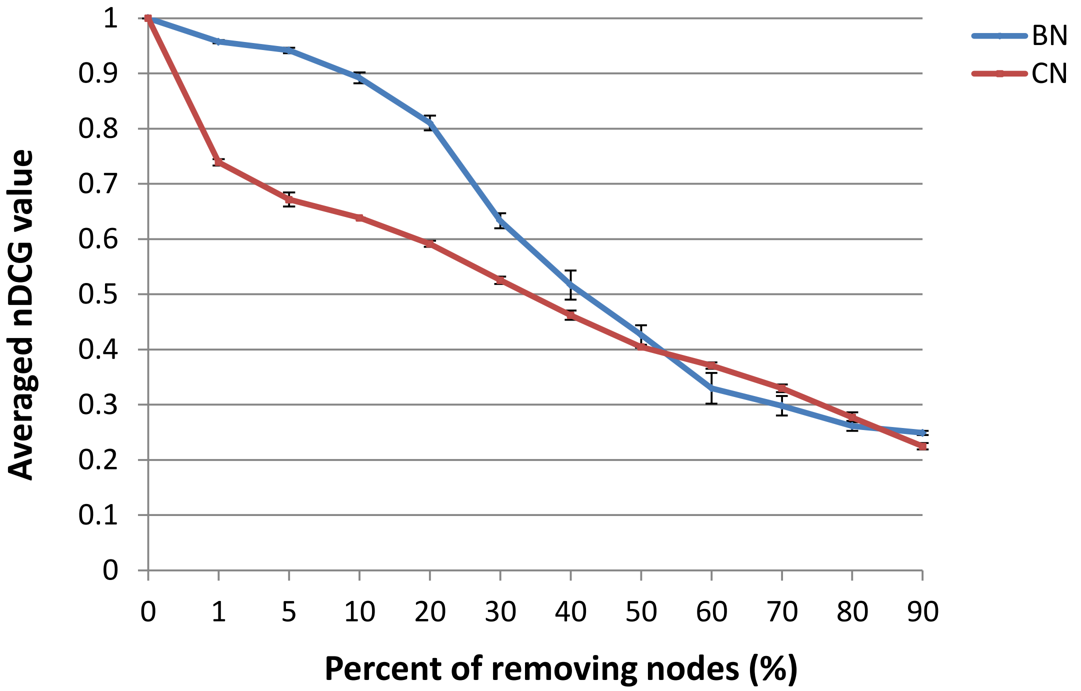

Supplementary Figure. 9: Permutation tests to assess the robustness of RACS. BN for the *background PPI network*, and CN for the *cancer network*.

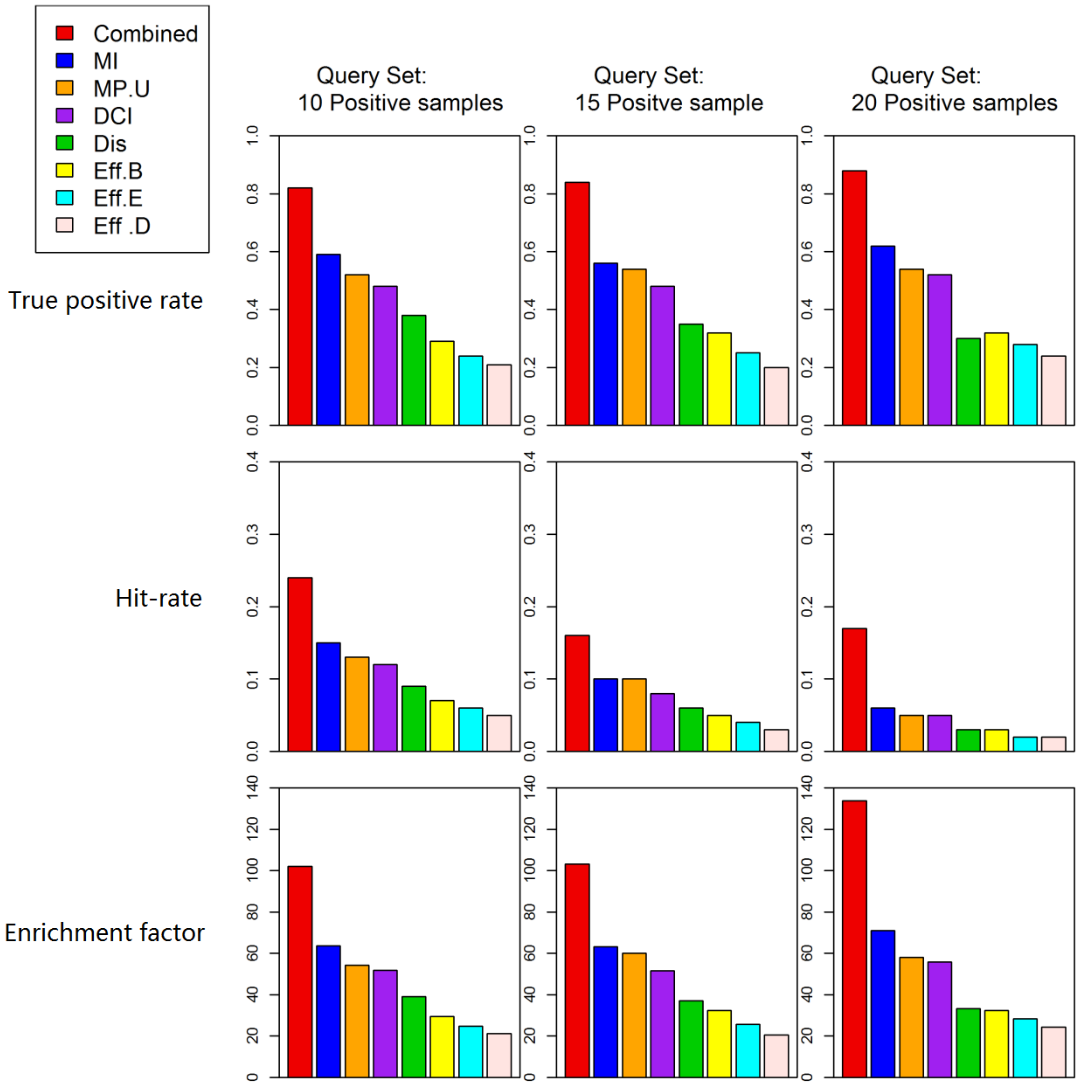

Supplementary Figure. 10: Assessment of the contribution of individual features and the combined features to model performance. Values are averaged for 10 times.

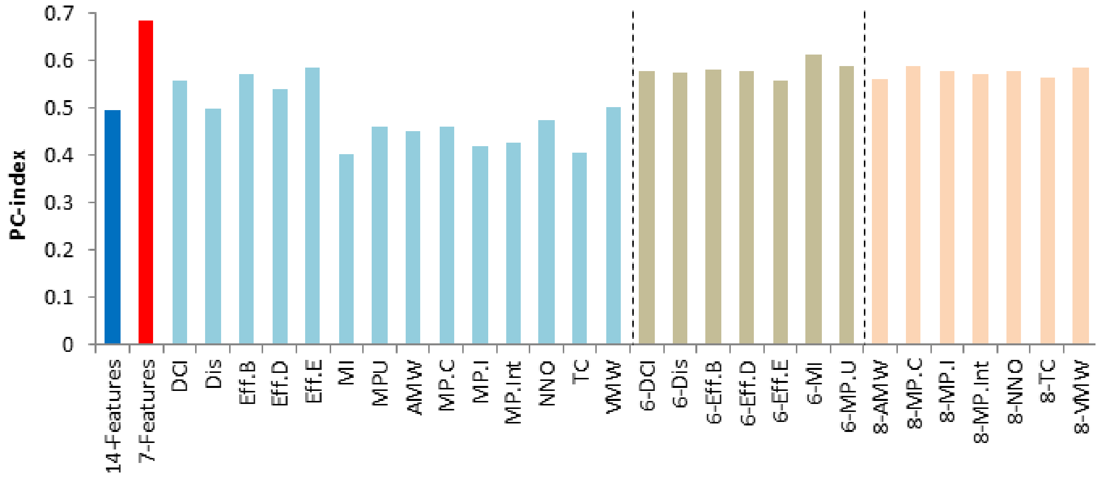

Supplementary Figure. 11: Performance of RACS with different choice of features. The light blue bars denote the performance of RACS using only one single feature, the dark gold ones denote the performance of RACS when one of the 7 significant feature was removed, while the pink bars represent the performance of RACS when one of the 7 insignificant feature was incorporated.

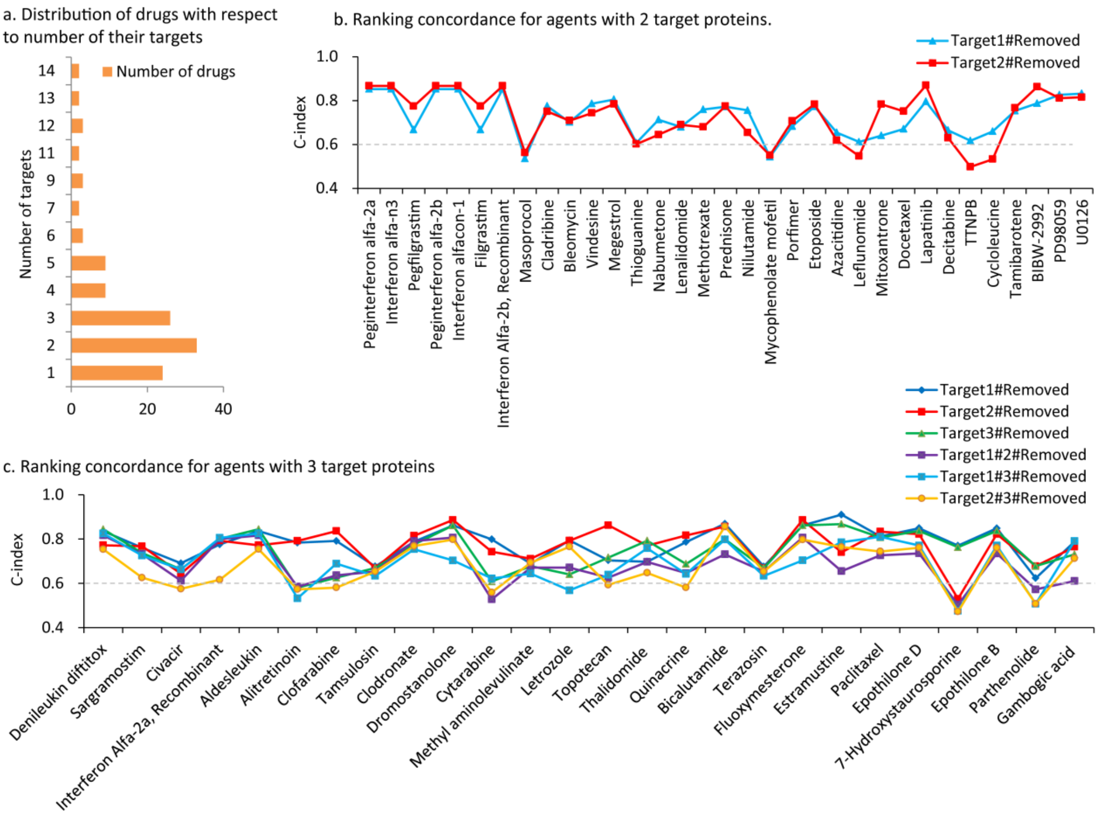

Supplementary Figure. 12: Evaluation of RACS performance by sub-selecting drug targets. a. Distribution of drugs with respect to number of their targets. b. Ranking concordance for agents with 2 target proteins. For each of the 33 drugs with 2 target proteins, 117 drug pairs were obtained when combined with the left 117 drugs. The concordance of predicted ranking lists of the 117 drug pairs before and after removing one of the targets were measured using C-index. c. Ranking concordance for agents with 3 target proteins. For each of the 26 drugs with 3 target proteins, the three single targets together with all three 2-targets-combinations were removed respectively each time. The concordance of predicted ranking lists of the 117 drug pairs before and after removing one/two of the targets were measured using C-index.

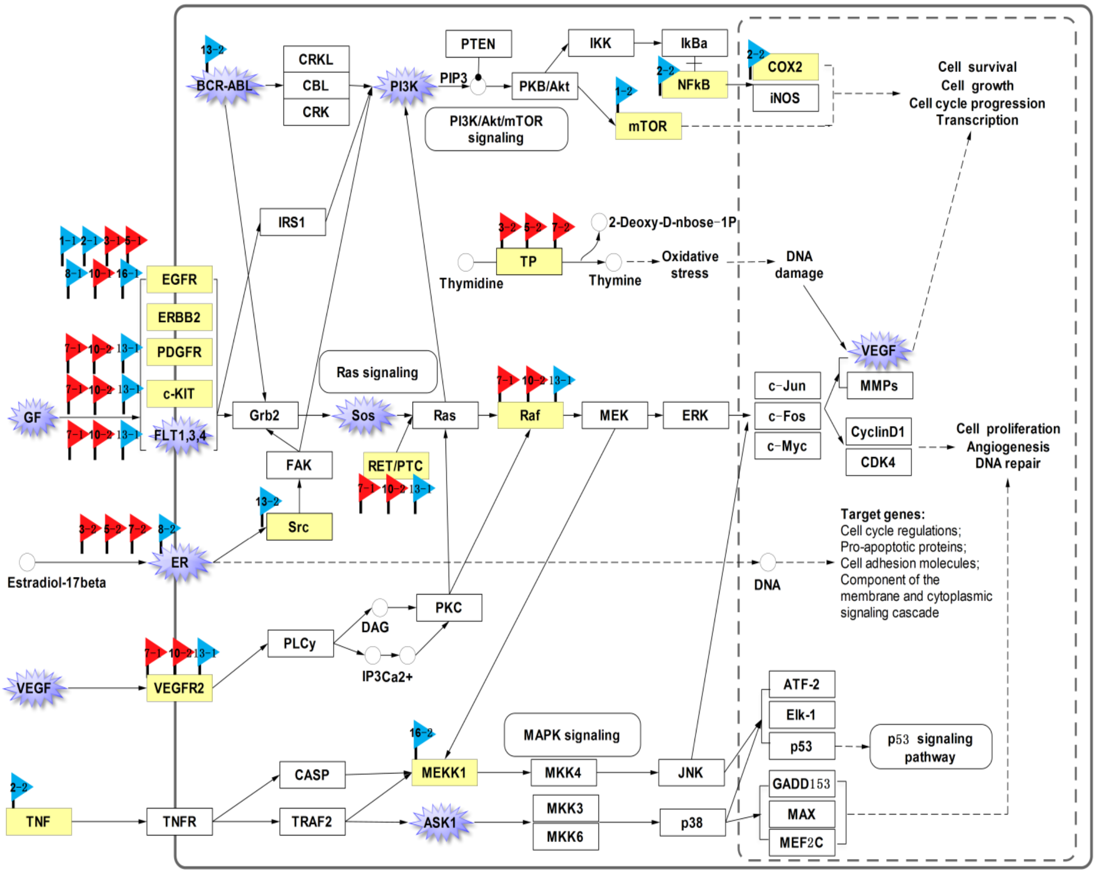

Supplementary Figure. 13: Targeting pathway of experimentally tested drug combinations in ER positive breast cancer cell MCF7. The genes within an explosion icon are those with mutation. The yellow ones were targets of the drugs tested in the MCF7 cell line experiment. The red, and blue flags mean this protein is targeted by drugs in the strong synergy drug pair, and synergy pair respectively. The number in the flag is consistent with the rank number of the drug pair in Fig. 3a, for example, for drug 1# in the pair with rank number 8, the flag would be labeled as 8-1, and similarly 8-2 for drug 2#.

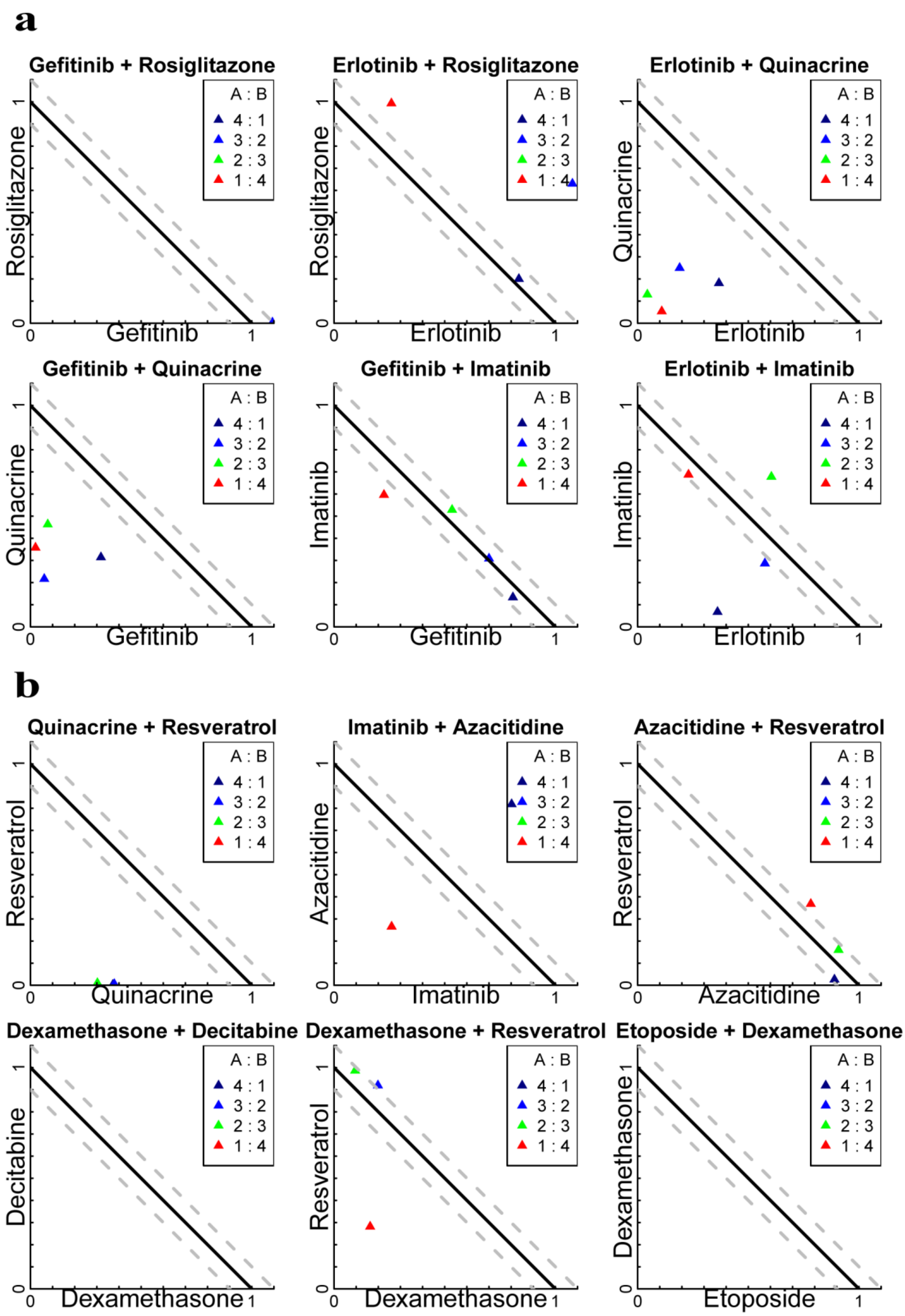

Supplementary Figure. 14: Isobolograms based on IC50s of the combination of different pairs on A549 cell lines. a. Isobolograms for the 6 top ranked agent pairs. b. Isobolograms for the 6 bottom ranked agent pairs. The horizontal axis and the vertical axis represent the relative dose of the drugs. The area between the two diagonal dotted gray lines in each plot represents the additive effect, while the triangular region under this additive effect area indicates

the synergy effect. Triangular points represent the relative concentrations of the combinations resulting in 50% inhibition of cancer cells. The two drugs were used alone or in combinations at 4 different concentration ratios: 4:1, 3:2, 2:3, and 1:4. Therefore, there were four Triangular points for each drug pair. The IC50s were not calculated for the pair of Decitabine and Dexamethasone because the inhibition ratios of each mixture were less than 30%, which means low sensitivity for A549 cell line**.**

Rank	Drug 1#	Drug 2#	CI (1# + 2#)				Result
			4+1	3+2	2+3	1+4	
1	Gefitinib (100uM)	Rosiglitazone (100uM)	1.50±0.16	1.79±0.14	1.87±0.04	2.46±0.06	
2	Erlotinib (100uM)	Rosiglitazone (100uM)	1.04±0.06	1.71±0.07	1.75±0.25	1.25±0.13	
3	Erlotinib (100uM)	Quinacrine (100uM)	0.55±0.07	0.44±0.04	**0.17±0.01**	**0.16±0.01**	
4	Gefitinib (100uM)	Quinacrine (100uM)	0.63±0.09	**0.28±0.06**	0.54±0.04	0.38±0.08	
5	Gefitinib (100uM)	Imatinib (100uM)	0.94±0.10	1.01±0.08	1.06±0.07	0.82±0.06	
6	Erlotinib (100uM)	Imatinib (100uM)	0.43±0.01	0.86±0.15	1.28±0.26	0.92±0.25	

Supplementary Figure. 15: Validation of the prediction result on NSCLC A549. Each drug with the corresponding maximum concentration used for the single drug in the experiment was recorded in **Drug1#** and **Drug2#**. The Chou and Talalay median-effect method was used to calculate the CI and summarized in a heat map. Green indicates synergy (CI < 0.9); yellow indicates additive (0.9 <CI <1.1); and red indicates antagonism (CI > 1.1). "4+1", "3+2", "2+3", and "1+4" indicate the two drugs were used in combinations at four different concentration ratios: 4:1, 3:2, 2:3, and 1:4. The Results summary table was rearranged according to the CI values in the order of "Strong synergy → Synergy → Additive effect → Antagonism".

Rank	Drug 1#	Drug 2#	CI (1# + 2#) 4+1	3+2	2+3	1+4	Result
50	Quinacrine (100uM)	Resveratrol (100uM)	0.37±0.07	0.38±0.05	0.31±0.06	1.54±0.07	
51	Imatinib (100uM)	Azacitidine (100uM)	1.62±0.08	1.88±0.09	1.42±0.03	0.53±0.08	
52	Azacitidine (100uM)	Resveratrol (100uM)	0.92±0.07	1.23±0.23	1.07±0.08	1.15±0.12	
53	Dexamethasone (100uM)	Decitabine (12.5uM)					
54	Dexamethasone (100uM)	Resveratrol (100uM)	8.06±0.06	1.12±0.04	1.08±0.01	0.44±0.13	
55	Etoposide (100uM)	Dexamethasone (100uM)	1.50±0.18	1.79±0.17	1.87±0.03	2.46±0.09	

Supplementary Figure. 16: Result of the validation experiment for the bottom ranked agent pairs on NSCLC A549. Each drug with the corresponding maximum concentration used for the single drug in the experiment was recorded in **Drug1#** and **Drug2#**. The Chou and Talalay median-effect method was used to calculate the CI and summarized in a heat map. Green indicates synergy (CI < 0.9); yellow indicates additive (0.9 <CI <1.1); and red indicates antagonism (CI > 1.1). “4+1”, “3+2”, “2+3”, and “1+4” indicate the two drugs were used in combinations at four different concentration ratios: 4:1, 3:2, 2:3, and 1:4.

a

#Animal Number: N=20/each group		Sorafenib (μM)								
Endpoints	Control	0.1	0.25	0.5	1	1.5	1.8	2	2.5	5
Cardiovascular toxicity	0	11/20	20/20	20/20	19/20	5/20				
Mortality rate	0				1/20	15/20	20/20	20/20	20/20	20/20

b

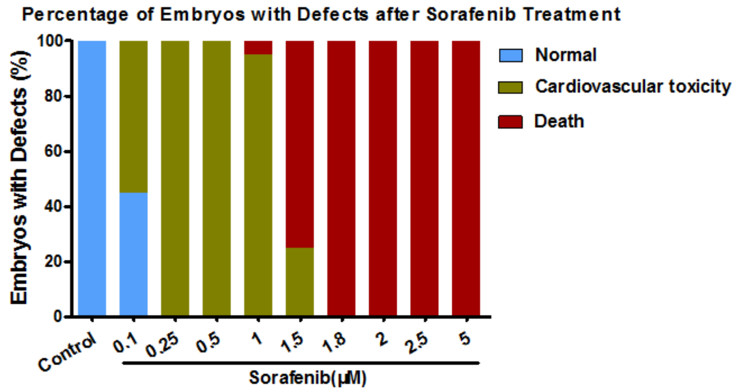

Supplementary Figure. 17: Maximum non-lethal concentration (MNLC) and LC50 determination of Sorafenib. a. statistic number of animal situations observed after Sorafenib treatment; b. graphic representation of the data from a.

a

#Animal Number: N=20/each group		Tamoxifen (μ M)												
Endpoints	Vehicle Control	0.1	0.25	0.5	1	2.5	4	5	7.5	10	20	40	60	80
Mortality rate	0	0	0	0	0	0	1/20	7/20	19/20	20/20	20/20	20/20	20/20	20/20

b

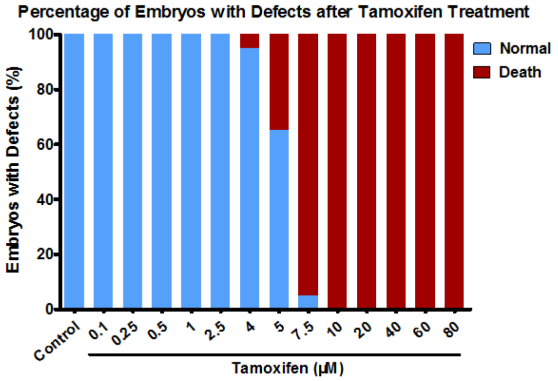

Supplementary Figure. 18: Maximum non-lethal concentration (MNLC) and LC50 determination of Tamoxifen. a. statistic number of animal situations observed after Tamoxifen treatment; b. graphic representation of the data from a.

a

Group	Treatment	Route	Number of Animals
1	Vehicle Control	Soaking	20
2	Erlotinib (8.4 μM) + Sorafenib (MNLC)	Soaking	20
3	Erlotinib (4.2 μM) + Sorafenib (MNLC)	Soaking	20
4	Sorafenib (0.6 μM) + Tamoxifen (MNLC)	Soaking	20
5	Erlotinib (12.6 μM) + Tamoxifen (MNLC)	Soaking	20
6	Gefitinib (2.52 μM) + Tamoxifen (MNLC)	Soaking	20
7	Gefitinib (1.68 μM) + Tamoxifen (MNLC)	Soaking	20

Note:
All compounds were dissolved in 0.1% DMSO (in fish water).
Vehicle Control: in 0.1% DMSO (in fish water);

b

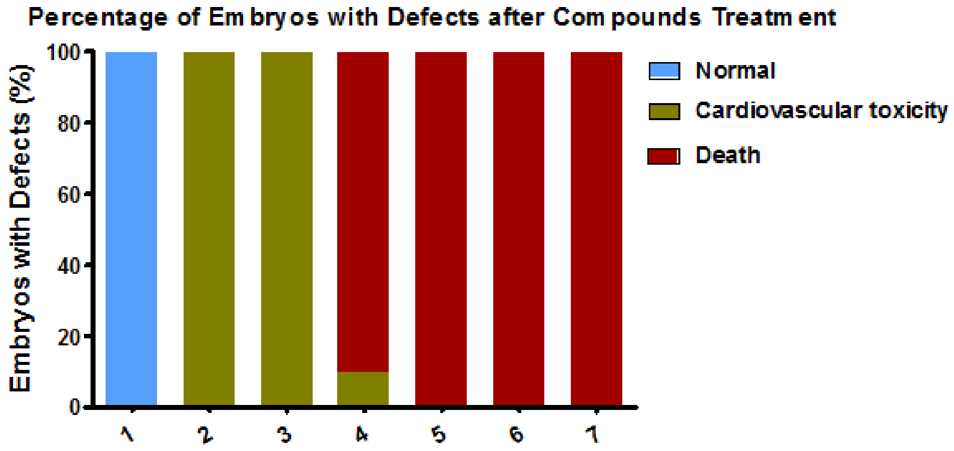

Supplementary Figure. 19: The toxicity of drug combination treatment on zebrafish development. a. the groups and treatment strategy; b. embryos situation after drug treatment.

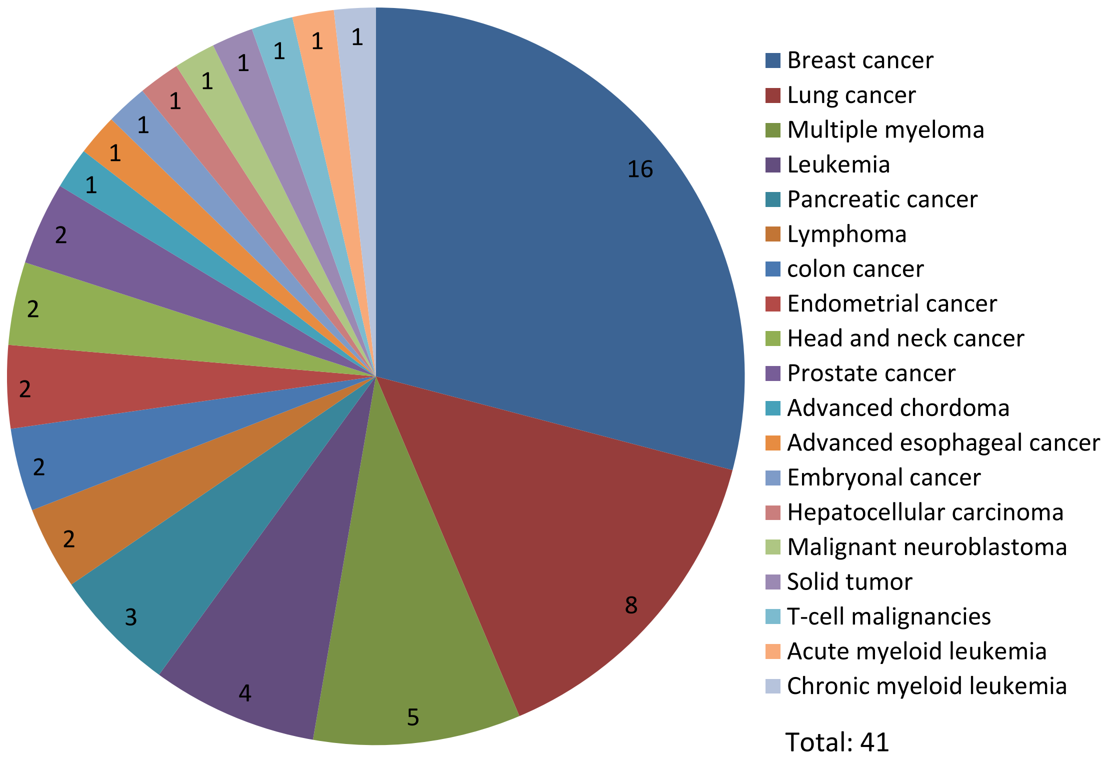

Supplementary Figure. 20: Number of synergistic agent pairs for each cancer type.

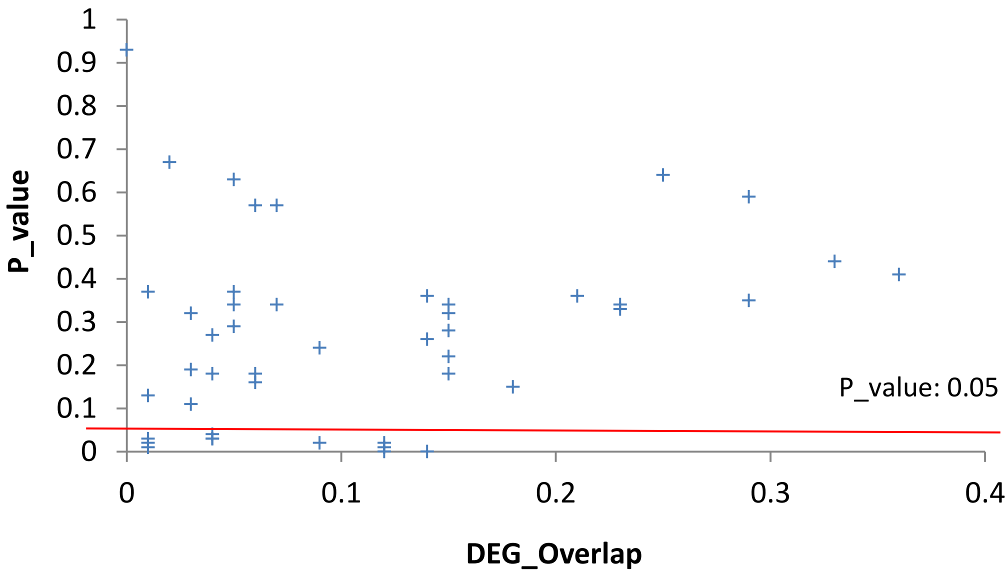

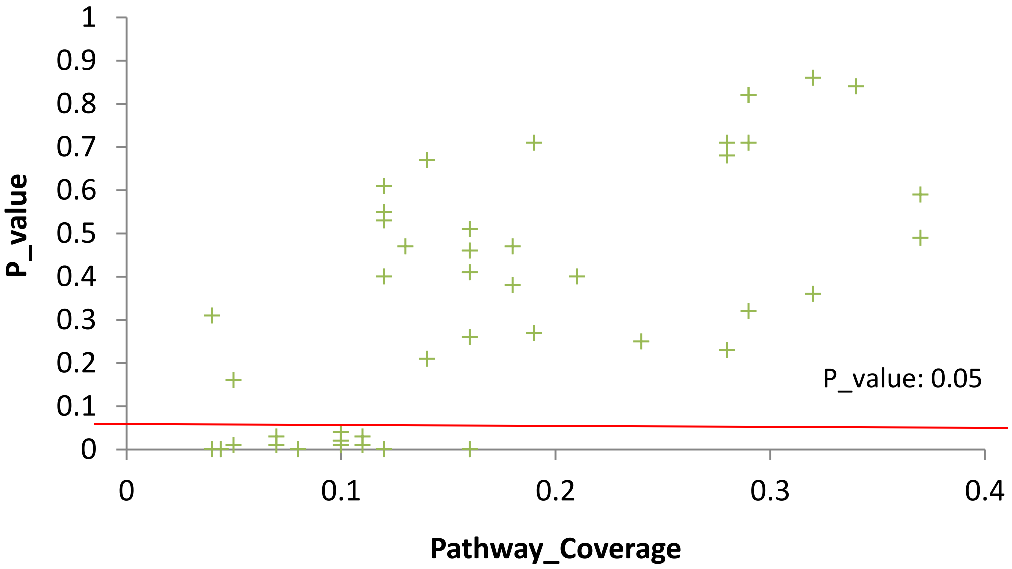

Supplementary Figure. 21: *DEG_Overlap* and *Pathway_Coverage* for the negative pairs. "+" denote the *P_value* of *DEG_Overlap* or *Pathway_Coverage* for each drug combinations.

Supplementary Tables

Supplementary Table 1: 14 features initially selected to differentiate synergistic pairs from unlabeled combinations.

No.	Feature	Indication	Select
1	*TC*	The similarity between the therapeutic categories (ATC code) of the two agents. *TC* was chosen to examine whether the two paired drugs in known synergistic combinations tend to share similar therapeutic effects.	×
2	*AMW*	The averaged molecular weight of the two agents. AMW was selected to see whether the drug components in the synergistic combinations tend to have small/big molecular weights.	×
3	*VMW*	The variance of molecular weight of the two agents. VMW was selected to see whether the drug components in the synergistic combinations tend to have similar molecular weights.	×
4	*MI*	The similarity between the biological processes (BPs) regulated by the targets of the two agents, and a large value often imply a high functional similarity. It was found that the paired drugs in the synergistic combinations tend to regulate different *cancer-related BPs*, as negative *MI* values were observed with the known synergistic combinations.	√
5	*DCI*	The variance between the combined agent pair-induced relative change in network information-transmitting efficiency and the sum of the change induced by the individual agents. Indicated by DCI, the synergistic combinations tend to produce more effects on reducing the information sending efficacy of the cancer network than the sum of the individual drugs.	√
6	*Dis*	The average distance between target proteins of the two agents in the context of PPI network. The drug components in the synergistic combinations tend to have shorter distance between target proteins in the context of protein-protein interaction network.	√
7	*Eff.D*	The evaluation of the efficacy of drug pairs considering both therapeutic effects and additional effects, calculated with degree of the drug target in the network. This feature was designed based on the assumption that good combinations are expected to generate maximum therapeutic effects and minimum additional effects.	√
8	*Eff.B*	The evaluation of the efficacy of drug pairs considering both therapeutic effects and additional effects, calculated with betweenness of the drug targets in the network. This feature was designed based on the assumption that good combinations are expected to generate maximum therapeutic effects and minimum additional effects.	√
9	*Eff.E*	The evaluation of the efficacy of drug pairs considering both	√

		therapeutic effects and additional effects, calculated with eigenvector centrality of the drug target in the network. This feature was designed based on the assumption that good combinations are expected to generate maximum therapeutic effects and minimum additional effects.	
10	*NNO*	The overlap rate between the network neighbors of the target sets of the two agents. This feature was used to see whether the paired drugs tend to share more interaction partners in the protein-protein interaction network.	×
11	*MP.I*	The proportion of identical pathways regulated by the targets of the two agents.	×
12	*MP.C*	The proportion of cross-talking pathways regulated by the targets of the two agents.	×
13	*MP.Int*	The proportion of interacting pathways regulated by the targets of the two agents.	×
14	*MP.U*	The proportion of unrelated pathways regulated by the targets of the two agents. We found the paired drugs in the synergistic combinations tend to regulate the unrelated *cancer-related pathways*.	√

Supplementary Table 2: 5 parameters derived from drug treated gene expression profile.

No.	Feature	Indication	Select
1	*DEG_Overlap*	The overlap rate of the differentially expressed genes of the two agents.	√
2	*DEG_n_Overlap*	The overlap rate of the network neighbors of the differentially expressed genes of the two agents.	×
3	*DEG_BP*	The similarity between the biologic processes of the differentially expressed genes of the two agents.	×
4	*Pathway_Coverage*	The coverage rate of the *breast cancer pathway* by the differentially expressed genes of the two agents.	√
5	*DEG_Dis*	The average shortest distance between the two differentially expressed gene sets of the two agents in PPI network	×

Supplementary Table 3: *DEG_Overlap* and *Pathway_Coverage* for the nine synergistic agent pairs.

No.	Drug_x [number of DEGs]	Drug_y [number of DEGs]	DEG_Overlap(x, y)	P_Value1	Pathway_Coverage(x, y)	P_Value1
1	Trastuzumab [776]	Tamoxifen [258]	0.06	0.02	0.09	< 0.01
2	Trastuzumab [776]	Bortezomib [4604]	0.12	1	0.32	0.37
3	Trastuzumab [776]	Paclitaxel [194]	0.04	0.01	0.08	< 0.01
4	Trastuzumab [776]	Doxorubicin [1376]	0.12	0.01	0.21	0.04
5	Trastuzumab [776]	Epirubicin [1909]	0.09	0.03	0.17	0.02
6	Doxorubicin [1376]	Paclitaxel [194]	0.14	0	0.16	0.04
7	Decitabine [109]	Paclitaxel [194]	0.02	0.04	0.02	0.03
8	Letrozole [547]	Lapatinib [1245]	0.11	0	0.12	0.03
9	Etoposide[943]	Tanespimycin[1120]	0.13	0.02	0.11	0.04
**P_Value2**			3.82e-006		4.62e-005	

Supplementary Table 4: Ranking result for the human β-cell lymphoma cell line Ly3 data (NCI-DREAM data).

Compound A	Compound B	DREAM Ranking	SynGen Ranking	DIGRE Ranking	DrugComboRanker Ranking	Zhao's method Ranking	RACS.Preliminary Ranking	RACS Ranking
Doxorubicin	H-7	1	3	18	45	70	12	12
Etoposide	H-7	2	1	38	55	37	22	19
Blebbistatin	H-7	3	32	19	14	66	58	44
Doxorubicin	Trichostatin A	4	8	26	33	36	3	3
Cycloheximide	H-7	5	65	13	25	19	49	36
Camptothecin	H-7	6	28	46	10	29	20	17
Etoposide	Trichostatin A	7	7	23	6	8	5	5
Cycloheximide	Monastrol	8	77	88	35	25	30	23
H-7	Monastrol	9	13	8	75	5	43	31
Camptothecin	Etoposide	10	44	47	2	2	1	1
Camptothecin	Doxorubicin	11	33	44	5	7	9	9
Monastrol	Trichostatin A	12	5	53	11	13	19	16
H-7	Trichostatin A	13	2	7	77	21	45	33
Monastrol	Vincristine	14	57	37	3	4	11	11
Blebbistatin	Monastrol	15	69	42	65	74	7	7
Aclacinomycin A	Monastrol	16	78	83	66	76	17	15
Doxorubicin	Etoposide	17	9	28	1	1	8	8
Methotrexate	Vincristine	18	50	73	22	24	2	2
Aclacinomycin A	H-7	19	74	2	56	78	24	20
H-7	Rapamycin	20	20	10	76	48	51	38
Geldanamycin	Vincristine	21	62	33	12	14	21	18
Blebbistatin	Vincristine	22	31	25	4	6	10	10

Geldanamycin	H-7	23	51	1	73	44	31	24
Etoposide	Vincristine	24	25	71	26	28	26	21
Aclacinomycin A	Cycloheximide	25	12	66	42	75	63	49
Methotrexate	Monastrol	26	72	55	7	9	4	4
H-7	Methotrexate	27	38	90	74	56	36	26
Cycloheximide	Trichostatin A	28	73	72	71	51	59	45
Cycloheximide	Vincristine	29	63	84	72	40	65	51
Blebbistatin	Geldanamycin	30	39	11	23	26	39	29
Methotrexate	Rapamycin	31	43	51	62	71	37	27
Doxorubicin	Vincristine	32	23	49	13	15	13	13
Rapamycin	Vincristine	33	27	14	53	59	14	62
H-7	Vincristine	34	18	16	78	42	48	35
Etoposide	Geldanamycin	35	52	4	48	55	29	22
Aclacinomycin A	Etoposide	36	70	15	61	69	47	34
Doxorubicin	Geldanamycin	37	47	81	58	63	15	14
Geldanamycin	Rapamycin	38	49	3	46	49	16	63
Monastrol	Rapamycin	39	6	20	16	17	25	66
Aclacinomycin A	Vincristine	40	64	65	24	27	60	46
Trichostatin A	Vincristine	41	30	45	50	57	62	48
Camptothecin	Vincristine	42	22	75	34	38	50	37
Blebbistatin	Methotrexate	43	29	74	47	50	66	52
Aclacinomycin A	Doxorubicin	44	68	63	20	22	35	25
Geldanamycin	Methotrexate	45	14	5	43	45	53	40
Methotrexate	Trichostatin A	46	58	40	57	62	56	42
Camptothecin	Trichostatin A	47	56	35	44	47	72	76
Blebbistatin	Rapamycin	48	40	21	28	30	34	71

Geldanamycin	Trichostatin A	49	66	58	27	54	41	30
Camptothecin	Cycloheximide	50	54	70	51	60	61	47
Rapamycin	Trichostatin A	51	4	22	21	23	38	28
Aclacinomycin A	Geldanamycin	52	11	79	15	16	32	69
Camptothecin	Methotrexate	53	37	89	29	31	44	32
Cycloheximide	Methotrexate	54	15	64	70	32	76	61
Aclacinomycin A	Rapamycin	55	71	6	18	20	23	65
Camptothecin	Monastrol	56	67	60	52	58	52	39
Cycloheximide	Doxorubicin	57	59	86	67	73	77	77
Cycloheximide	Etoposide	58	61	62	68	52	73	58
Camptothecin	Geldanamycin	59	48	78	54	61	64	50
Blebbistatin	Doxorubicin	60	35	48	9	12	54	41
Doxorubicin	Methotrexate	61	45	56	8	11	68	54
Aclacinomycin A	Blebbistatin	62	42	91	63	3	28	68
Geldanamycin	Monastrol	63	76	82	36	39	46	74
Etoposide	Methotrexate	64	46	77	38	10	70	56
Aclacinomycin A	Methotrexate	65	16	17	37	53	71	57
Cycloheximide	Rapamycin	66	60	69	19	46	78	78
Blebbistatin	Etoposide	67	36	57	17	18	67	53
Blebbistatin	Trichostatin A	68	53	61	59	64	55	75
Cycloheximide	Geldanamycin	69	10	59	69	67	74	59
Etoposide	Rapamycin	70	19	39	30	33	27	67
Aclacinomycin A	Trichostatin A	71	75	85	39	77	6	6
Blebbistatin	Cycloheximide	72	41	87	49	68	57	43
Aclacinomycin A	Camptothecin	73	55	80	64	72	69	55
Doxorubicin	Rapamycin	74	21	36	41	43	40	72

Blebbistatin	Camptothecin	75	17	68	40	41	75	60
Camptothecin	Rapamycin	76	34	50	32	35	33	70
Etoposide	Monastrol	77	24	41	31	34	18	64
Doxorubicin	Monastrol	78	26	43	60	65	42	73

Supplementary Table 5: Known synergistic anti-cancer drug combinations.

NO.	Agent_A	Agent_B	
1	Arsenic trioxide	Tanespimycin(17-Aag)	*
2	Gefitinib	Irinotecan	
3	Etoposide	Tanespimycin(17-Aag)	*
4	Erlotinib	Pemetrexed	*
5	Bortezomib	Vorinostat	*
6	Bortezomib	Lonafarnib	
7	Trastuzumab	Tamoxifen	*
8	Gefitinib	PD98059	*
9	Tanespimycin(17-Aag)	U0126	*
10	Trastuzumab	Bortezomib	*
11	Gleevec	Vorinostat	*
12	Paclitaxel	Lonafarnib	
13	Paclitaxel	Tubacin	
14	Paclitaxel	Trichostatin A	*
15	Decitabine	Depsipeptide	
16	Retinoic Acid	Trichostatin A	
17	Doxorubicin	Paclitaxel	
18	Bortezomib	Lenalidomide	*
19	Rituximab	Fludarabine	*
20	Cetuximab	BIBW-2992	*
21	Trastuzumab	Paclitaxel	*
22	Zoledronic acid	Doxorubicin	
23	Toremifene	Paclitaxel	
24	Paclitaxel	2-Methoxyestradiol	*
25	Irinotecan	Docetaxel	
26	Bortezomib	Etoposide	*
27	Bevacizumab	Erlotinib	*
28	Paclitaxel	Decitabine	*
29	Rosiglitazone	Carboplatin	
30	Sorafenib	Carboplatin	
31	LY294002	Roscovitine	*
32	Sorafenib	Genistein	*
33	Bevacizumab	Paclitaxel	*
34	Lenalidomide	Dexamethasone	*
35	Gleevec	Sirolimus	*
36	Trastuzumab	Carboplatin	
37	Trastuzumab	Vinorelbine	*
38	Trastuzumab	Doxorubicin	
39	Trastuzumab	Epirubicin	
40	Letrozole	Lapatinib	*
41	Topotecan	Lapatinib	*

"*" the finally selected 26 positive samples used to build the prediction model.

Supplementary Table 6: 41 top-ranked agent pairs for ER positive breast cancer cell line MCF7.

No.	Drug_A_Name (concentration)	Drug_B_Name (concentration)	Details
1	Gefitinib (42μM)	BIBW-2992 (30nM)	To be validated by cell-line experiment
2	Gefitinib (42μM)	Tamoxifen (1000μM)	To be validated by cell-line experiment
3	Gefitinib (42μM)	Everolimus (20μM)	To be validated by cell-line experiment
4	Gefitinib (42μM)	Sorafenib (30μM)	To be validated by cell-line experiment
5	Gefitinib (42μM)	Thalidomide (300μM)	To be validated by cell-line experiment
6	Gefitinib (42μM)	Erlotinib (210μM)	To be validated by cell-line experiment
7	Gefitinib (42μM)	Toremifene (200μM)	To be validated by cell-line experiment
8	Gefitinib (42μM)	PD98059 (240μM)	To be validated by cell-line experiment
9	Erlotinib (210μM)	Flavopiridol (4800nM)	To be validated by cell-line experiment
10	Erlotinib (210μM)	Tamoxifen (1000μM)	To be validated by cell-line experiment
11	Erlotinib (210μM)	Sorafenib (30μM)	To be validated by cell-line experiment
12	Erlotinib (210μM)	Sunitinib (297.68μM)	To be validated by cell-line experiment
13	Sorafenib (30μM)	Tamoxifen (1000μM)	To be validated by cell-line experiment
14	Sorafenib (30μM)	Dasatinib (840μM)	To be validated by cell-line experiment
15	Sorafenib (30μM)	Everolimus (20μM)	To be validated by cell-line experiment
16	Everolimus (20μM)	BIBW-2992 (30nM)	To be validated by cell-line experiment
17	Tamoxifen (1000μM)	Flavopiridol (4800nM)	To be validated by cell-line experiment
18	Topotecan	Vorinostat	Not commercially available
19	Isotretinoin	Cytarabine	Not commercially available
20	Vorinostat	Arsenic trioxide	Not commercially available
21	Gambogic acid	Docetaxel	Not commercially available
22	Everolimus	Etoposide	Not commercially available
23	Toremifene	Fulvestrant	Not commercially available
24	Evodiamine	Parthenolide	Not commercially available
25	Trastuzumab	BIBW-2992	With antibody
26	Erlotinib	Panitumumab	With antibody
27	Cetuximab	Gefitinib	With antibody
28	Trastuzumab	Letrozole	With antibody
29	Curcumin	Resveratrol	Literature_supported (colorectal cancer)
30	Trastuzumab	Erlotinib	Literature_supported (breast cancer)
31	Topotecan	Vorinostat	Literature_supported (small cell lung cancer)
32	Estramustine	Docetaxel	Literature_supported (breast cancer)

33	Bleomycin	Etoposide	Literature_supported (endometrial carcinoma)
34	Letrozole	BIBW-2992	Deleted by filtering parameters of step 3
35	Pemetrexed	Mitotane	Deleted by filtering parameters of step 3
36	Pamidronate	Ifosfamide	Deleted by filtering parameters of step 3
37	Zoledronate	Mitoxantrone	Deleted by filtering parameters of step 3
38	Topotecan	Leflunomide	Deleted by filtering parameters of step 3
39	Thioguanine	Mercaptopurine	Deleted by filtering parameters of step 3
40	Vindesine	Epothilone D	Deleted by filtering parameters of step 3
41	Epothilone B	2-Methoxyestradiol	Deleted by filtering parameters of step 3

Concentration: this is the maximum concentration used for each single drug in the cell line experiment

Supplementary Table 7: Array information of the 11 agents on human breast cancer cell line MCF7.

Drug_Name	Data_Source	DataSet_ID	Platform
Bortezomib	GEO	GSE8565	HG-U133_Plus_2
Decitabine	CMAP	920	HT_HG-U133A_EA
Doxorubicin	CMAP	3291	HT_HG-U133A
Epirubicin	GEO	GSE39042	HG-U133_Plus_2
Etoposide	CMAP	1626	HG-U133A
Lapatinib	ArrayExpress	E-MEXP-440	HG-U133A
Letrozole	CMAP	4824	HT_HG-U133A
Paclitaxel	CMAP	640	HG-U133A
Tamoxifen	CMAP	2212	HT_HG-U133A
Tanespimycin	CMAP	1147	HT_HG-U133A
Trastuzumab	GEO	GSE15043	HG-U133_Plus_2

Supplementary Table 8: Array information of the 11 agents on human lung cancer cell line A549.

Drug_Name	Data_Source	DataSet_ID	Platform
Azacitidine	GEO	GSE29077	HG-U133A_2
Dasatinib	ArrayExpress	E-TABM-585	HG-U133A
Decitabine	GEO	GSE29077	HG-U133A_2
Dexamethasone	GEO	GSE17307	HG-U133_Plus_2
Erlotinib	GEO	GSE57422	HuGene-2_1-st
Etoposide	ArrayExpress	E-MIMR-102	HG_U95Av2
Gefitinib	GEO	GSE4342	HG-U133A
Imatinib	ArrayExpress	E-TABM-585	HG-U133A
Quinacrine	GEO	GSE57422	HuGene-2_1-st
Resveratrol	GEO	GSE9008	HG-U133_Plus_2
Rosiglitazone	GEO	GSE7035	HG-U133A

Supplementary Table 9: Growth inhibition rates of Everolimus and BIBW2992 under six concentration gradients in human breast cancer cell line MCF7.

Concentration gradient*	Growth inhibition rates of Everolimus (20μM)	Growth inhibition rates of BIBW2992 (30nM)
**1**	77.76	20.43
**2**	52.49	6.34
**3**	45.17	8.14
**4**	40.75	6.94
**5**	39.15	2.60
**6**	31.20	1.20

*1-6： Each single drug was diluted 1:4 with 10% PBS into six concentration gradients.

Supplementary Note 1: Cell line experiment for breast cancer

Agents**:** Dasatinib, erlotinib, everolimus, gefitinib, sorafenib, sunitinib, azacitidine, cycloleucine, cytarabine, decitabine, etoposide, genistein, ifosfamide, melatonin, parthenolide, resveratrol, tamibarotene, terazosin, tretinoin, and tamoxifen were purchased from Biovision (Mountain View, CA). BIBW-2992, flavopiridol, PD98059, thalidomide, lapatinib, and toremifene were purchased from Selleckchem (Houston, TX, USA). The purity of each drug is above 98%.

Cell line**:** Human breast cancer cell line MCF7 was obtained from American Tissue Type Culture Collection (ATCC, Rockville, MD). Cells were maintained in a humidified 37°C atmosphere containing 5% CO2 and cultured in Dulbecco's modified Eagle's medium (DMEM) supplemented with 10% fetal bovine serum.

MTT assay**:** The cytotoxicity of each drug or combination was evaluated by MTT assay. The cells were seeded in 96-well flat-bottomed plates at a density of 8000 cells/well in 100 μl complete medium overnight. The medium was then discarded, and fresh medium containing various drugs or their combination were added at indicated concentrations and left in contact for 72 h. After that, 10 μl MTT (5 mg/mL; Sigma, St. Louis, MO) was added to each well, and the plates were incubated for 4 h at 37°C. Then, 100 μl formazan dissolving solution (20% SDS in 50% N, N-dimethylformamide, containing 0.5% [v/v] 80% acetic acid and 0.4% [v/v] 1 M HCl) was added to each well for 4 h. The optical density (proportional to the number of live cells) was assessed using a microplate reader (Tecan, Research Triangle Park, NC, USA) at 570 nm.

***Drug treatment*:** Drugs were dissolved in DMEM medium containing 10% PBS. For each drug pair, blank control groups (PBS only), as well as control groups (cells treated with DMEM with 10% PBS) were run with the assays and produced no cytotoxic effects. The two drugs were used alone or in combinations at 4 different concentration ratios: 4:1, 3:2, 2:3, and 1:4. To calculate the half inhibitory concentration (IC_{50}), the single drug or the combined drugs at the four different concentration ratios were diluted 1:4 with 10% PBS into six concentration gradients. For each concentration gradient, there were three replicates, and each experiment was repeated three times.

***IC_{50}*:** Chemosensitivity was expressed as the IC_{50} value. The IC_{50} values were calculated by the following formula:

$$IC_{50} = 10^{[x_m - i(\sum P - 0.5)]}$$

Where x_m is the log value for the maximal concentration of the agent, i is the log value for the dilution ratio, which is 4 in this validation experiment, ΣP is the sum of the six growth inhibition rates of each drug-treated group, and 0.5 is the empirical constant.

***The combination index*:** The combination index (*CI*) defined by Chou and Talalay was introduced to determine whether a combined drug pair could produce synergy:

$CI=a/A+b/B$

where A and B are the IC_{50} of agent x and agent y, respectively, and a and b are the IC_{50} when the two agents are combined.

Generally, it is considered that a CI value <0.9 indicates synergism, $0.9 < CI < 1.1$ indicates an additive effect, and $CI > 1.1$ indicates antagonism. Here, an agent pair was recognized as

synergistic if all of the four *CI* values calculated from the four different concentration ratios were < 0.9.

Supplementary Note 2: Evaluating performance of the preliminary ranking model

The prediction performance of the preliminary ranking model was assessed using the 26 labeled samples and the pairwise drug combinations of the 118 anti-cancer drugs (unlabeled samples). Ten cross-validations were performed using 10, 15, or 20 positive samples as a training set. The rest labeled samples together with the unlabeled samples were used as the testing set. The model performance was evaluated by the true positive rate, hit-rate, and enrichment-factor within the top-1% list for all cross-validations (Supplementary Fig. 5). As a result, the true positive rate of positive samples ranged from 0.73~1.00, with a mean of 0.84, indicating the ability to rank the true positives on the top list. The mean hit-rate was 0.17 (0.09~0.28). This is not unexpected as the hit-rate is generally influenced by the density of the positive samples in the testing dataset. Strikingly, the enrichment-factor achieved a mean of 113.06 times comparing to a random distribution, suggesting the remarkable ability of our model to concentrate positive pairs to the top ranking list.

At the same time, more data with fewer features were tested in this paper. When *MP.U* and *Eff.E* were excluded, 10,011 pairs (142 drugs) containing 41 positive samples could be retrieved. Ten cross-validations were performed using 10, 20, or 30 positive samples as a training set. Results of the true positive rate of positive samples ranged from 0.71~1.00 with mean of 0.81

(Supplementary Fig. 6), slightly worse than that of the model using more features with fewer data. This may be related to the function of the semi-supervised model, which ranks potential samples better based on the more number of significant features.

This model of manifold ranking was also compared with one-class SVM, a commonly used classification model when only positive samples are available[1]. The same training data and testing data from the preliminary ranking model were kept. The results (Supplementary Fig. 7) indicated that the manifold ranking model outperforms one-class SVM by improving 48.75% in true positive rate, 119.39% in hit-rate, and 118.30% in enrichment-factor.

Performance evaluation

True positive rate: the recall ratio of positive samples in the top-1% list of the prediction results.

$$True\ positive\ rate = \frac{m}{\mathrm{n}}$$

Hit-rate: the ratio of positive samples in the top-1% list.

$$Hit - rate = \frac{m}{\mathrm{M}}$$

Enrichment-factor: the magnitude of hit-rate improvement over random selection of positive samples from all agent pairs.

$$Enrichment - factor = \frac{m/M}{n/N}$$

m: number of positive samples in the top-1% list of the prediction result;

n: number of positive samples in the testing set;

M: number of samples in the top-1% list;

N: number of samples in the testing set.

Supplementary Note 3: Robustness of RACS

The robustness of RACS was associated with the *background PPI network* and the *cancer network*, as the discriminating features used in preliminary ranking model were calculated based on these networks. To test the robustness of RACS according to the perturbation of the networks, protein nodes in the *background PPI network* or the *cancer network* were randomly removed at different percentages for 3 times, to investigate the influence on the values of the discriminating features respectively. As a result, all the features remained discriminating (with |Z-score|>3) until more than 10% of nodes were randomly removed in the networks (Supplementary Fig. 8). Next, these permutated features were incorporated into RACS to obtain the ranking list on the dataset used in "Evaluating performance of the preliminary ranking model" part. The normalized Discounted Cumulative Gain (nDCG: http://en.wikipedia.org/wiki/NDCG) was employed to measure the similarity between the permutation ranks and the original one with one query set containing 10 positive samples. The permutation rank results showed that the performance of the preliminary ranking model kept acceptable (with nDCG value ~ 0.6) until more than 20% of the nodes were removed from the two networks respectively (Supplementary Fig. 9). It was reasonable that random removing of a small number of nodes made no big change of the network as the majority of the nodes were of less connection with other nodes, while the removing of a large amount of nodes made the network completely different.

Supplementary Note 4: Contribution of individual features

The contribution of the individual features was further evaluated by different query set of the model on ER positive breast cancer data. As shown in Supplementary Fig. 10, the two functional features of GO-based mutual information entropy (*MI*) and the unrelated mapped pathway (*MP.U*) always gave better results than the remaining 5 network targeting features in terms of the pick-up rate, hit-rate, and enrichment-factor, suggesting that the functional interference is more indicative of synergistic effects. Despite the varied performance of individual features, calculations based on combined features can significantly improve the results more than 2-fold of that of any individual feature.

Supplementary Note 5: RACS performed best when combining the 7 significant features

Fourteen features covering chemical structure, pharmacology, functional and network properties of drug targets were initially designed in our study. Seven of them were identified as significant in differentiating synergistic pairs from unlabeled combinations. These 7 features might overlapped/correlated with each other, and also, the features that showed insignificant alone may have synergistic effects in the model once incorporated. To rule out such situation, we tested the performance of RACS on the DREAM data of 13 drugs with target protein information by cutting down one of the 7 significant features or increasing one of the 7 insignificant features. Generally, RACS performed better using the 7 single significant features than using the insignificant features (Supplementary Fig. 11). RACS obtained a PC-index of 0.69 when

combining the 7 significant features together, while the PC-index of RACS decreased to 0.49 when all 14 features were used (Supplementary Fig. 11). Among the 7 models using only 6 of the significant features, the one with MI removed performed best. But, none of them surpassed the RACS model with all the 7 significant features (Supplementary Fig. 11). Besides, the model performance worsened when incorporating one of the 7 insignificant features with the significant features (Supplementary Fig. 11).

Supplementary Note 6: Performance of RACS when sub-selecting the drug targets

RACS model was constructed by combining knowledge derived from the target proteins and the gene expression profiles of drug response. Currently, the target protein information of lots of compounds has been accumulated. But in the meantime, target proteins have not been completely known for many compounds. Given that this may bias the prediction accuracy of RACS, we further evaluated the performance of RACS by sub-selecting the target proteins.

The 118 collected drugs target 241 human proteins in total. Most of these drugs target only a few proteins (1~3), while some have many targets. For example, Gleevec® has 11 targets and Arsenic trioxide has 13 targets. As drugs with 2 or 3 targets account for most of the multi-target drugs (Supplementary Fig. 12), these drugs were chosen to show how RACS would perform when a subset of the target proteins were missed. For each of these drugs, 117 drug pairs were obtained when combine with the left 117 drugs. The concordance of predicted rankings of the 117 drug pairs before and after sub-selecting the target proteins were measured using C-index. For the drugs with 2 targets, the two targets were removed respectively in every simulation. As a result,

for most drugs, the predicted ranking list after removing one target showed high concordance (with C-index>0.7) with the original ranking (Supplementary Fig. 12). For the drugs with 3 targets, the three single targets together with all three 2-targets-combinations were removed respectively each time. Similar high concordance was seen when one of the targets were removed from most of the drug with 3 targets, while the concordance became a little lower when two of the targets were removed (Supplementary Fig. 12).

Supplementary Note 7: The construction of "*Targeting pathway of experimentally tested drug combinations in ER positive breast cancer cell MCF7*"

First, pathways closely related to breast cancer were collected, i.e., Estrogen Signaling pathway, ERBB pathway, PI3K/Akt/mTOR Signaling Pathway, p53 Signaling Pathway, Ras Signaling Pathway, Notch Signaling Pathway, Wnt Signaling pathway and NFkB Pathway. These pathways were compiled into a big pathway via the overlapping elements. Next, this pathway was modified using gene expression profiles and mutation information of MCF7 in CCLE[2] to make this pathway specific to MCF7 cell line. Drug targets were then mapped to the pathway, and for better display, pathways not targeted were removed to form the final "*Targeting pathway of experimentally tested drug combinations in ER positive breast cancer cell MCF7*".

Supplementary Note 8: Cell line experiment for lung cancer

Agents: Azacitidine, Dasatinib, Decitabine, Erlotinib, Etoposide, Gefitinib, Quinacrine, Resveratrol and Rosiglitazone were purchased from Biovision (Mountain View, CA). Dexamethasone and Imatinib were purchased from Selleckchem (Houston, TX, USA). The purity of each drug is above 98%.

Cell line: NSCLC cell lines A549 was obtained from the Shanghai Cell Bank, Chinese Academy of Sciences. Cells were grown in F12K mediums supplemented with 10% fetal serum and 100 μg/ml penicillin and 100 μg/ml streptomycin at 37℃ under 5% CO_2.

MTT assay: Each well of 96-well plates was seeded with 100 μl of cell suspension containing 5000 cells, leaving 100μl room for testing drugs. Drugs were added next day after seeding. The plates were continuously cultured for another 48 hours. The cytotoxicity was assayed by MTT method[3]

Isobologram: The anti-tumor effects of any two of eleven drugs combination were analyzed by isobolograms[4, 5]. Appropriate highest concentrations (100μM) of any two drugs ([A] and [B]) were set for each cell line. A/B mixtures = 5/0, 4/1, 3/2, 1/4 and 0/5 were serially diluted and added into wells, respectively. The cytotoxicity of the combinations, A or B alone and control was assayed by MTT methods. The isobolograms were established based on the IC_{50} data. The combination index (CI) was calculated as follows: CI= IC50 of A in mixture/ IC50 of A alone + IC50 of B in mixture/ IC50 of B alone. If all of the CIs except 0/5 and 5/0 were < 0.9, the combined effect was

synergistic; > 1.1 the combination was antagonistic; and with 0.9<= CI <=1.1, the combination was additive.

Supplementary Note 9: Validation of drug synergy and potential toxicity in vivo

Chemicals and reagents: Chemicals and compounds were purchased from Sigma-Aldrich Co.(St. Louis, MO) and Selleckchem unless otherwise specified. Cell culture reagents were from Invitrogen (Carlsbad, CA) or American Type Culture Collection (ATCC, Manassas, VA). Cell Tracker CM-DiI was purchased from Invitrogen.

Zebrafish Care and Maintenance: Zebrafish were raised and maintained under standard conditions[6]. Embryos were staged according to the work of Kimmel et al.[7]. The establishment and characterization of the fli1a-EGFP; Casper transgenic lines has been described elsewhere[8, 9]. The zebrafish facility at Shanghai Research Center for Model Organisms is accredited by the Association for Assessment and Accreditation of Laboratory Animal Care (AAALAC) International. All animal experiments were approved by the Institutional Animal Care and Use Committee of Shanghai Research Center for Model Organisms (IACUC NO.2015-0012).

Maximum non-lethal concentration (MNLC) and LC50 determination: To determine the MNLC and LC50 of lead compounds, zebrafish were treated from 2-dpf to 5-dpf and mortality was recorded every 24 h. Dead zebrafish was defined as the absence of heartbeat under a dissecting stereomicroscope (Nikon SMZ645; Japan). In the initial tests, five concentrations (0.1,

0.25, 1, 2.5, and 5mM/L) were used for compounds. If MNLC and LC50 could not be found from the initial tests, additional concentrations within the range of 0.01–80mM/L were further tested. Mortality curves were generated using GraphPad Prism 5.0 (GraphPad Software, San Diego, CA, USA) and the MNLC was determined with logistic regression.

Zebrafish Tumor Cell Xenograft: Zebrafish embryos were obtained using standard mating conditions and staged for cell xenotransplantation at 48 h post fertilization. After staining of cancer cells, embryos were dechorionized using micro-forceps and anesthetized with 0.0016% tricaine and positioned on their right side on a wet 1.0% agarose pad. Tumor cells were detached from culture dishes using 0.05% trypsin-EDTA and washed twice with PBS at room temperature. Cells were stained with 5μg/ml CM-DiI diluted in PBS and washed four times: once with FBS, twice with PBS and then once with 10% FBS diluted in PBS. Cells prepared following this procedure were: (a) ⩾90% viable, as assessed by exclusion of TRYPAN blue dye, (b) uniformly stained red and highly fluorescent, (c) in an easily injectable single cell suspension. Cells were kept on ice before injection. Approximately 200 cancer cells were injected into the yolk sac. Injected embryos were transferred to a 6-well plate (BD Falcon) containing drug of interest diluted in 5ml fresh fish water and maintained at 31℃for up to 4 dpi when the experiments were terminated and animals were euthanized by overexposure to tricaine.

Image acquisition: Embryos and larvae were analyzed with Nikon SMZ 1500 Fluorescence microscope and subsequently photographed with digital cameras. Quantitative image analyses were performed using image based morphometric analysis (NIS-Elements D3.1, Japan). A subset

of images was adjusted for levels, brightness, contrast, hue and saturation with Adobe Photoshop 7.0 software (Adobe, San Jose, California) to optimally visualize the expression patterns.

Statistical analysis: All data are presented as mean ± SEM. Statistical analysis and graphical representation of the data were performed using GraphPad Prism 5.0 (GraphPad Software, San Diego, CA). Statistical significance was performed using a ANOVA or $\chi 2$ test as appropriate. Statistical significance is indicated by *, where $P < 0.05$, and **, where $P < 0.01$, and ***, where $P < 0.0001$.

Supplementary Note 10: Networks for feature/parameter selection

Different networks were built to calculate different features: (i) The *background PPI network*: whole PPI data from online PPI databases (HPRD[10], Mint[11], Intact[12], BioGRID[13], DIP[14], MIPS[15]) were downloaded to construct a comprehensive background network. Then, the biggest connected component of this network was extracted as the final *background PPI network* with all interactions treated equally in the context of the PPI network. (ii) The *cancer network:* proteins in '*Pathways in cancer*' as well as other cancer-related proteins was firstly obtained as a network core from KEGG PATHWAY database[16] and literatures. Then their partner proteins in the context of PPIs were added to form an extended *cancer network*. (iii) The *specific cancer pathway* (taking breast cancer MCF7 as an example): gene expression profiles of cancer cell lines were obtained from CCLE. The differentially expressed genes in MCF7 were selected based on the fold-change of gene expression between MCF7 cell line and the average of expression value of all the other cell lines under the cutoff of |log2foldchange|>0.5. Then, these differentially expressed genes were connected via background PPI network to form the *breast cancer pathway*.

Supplementary Note 11: Four pathway interaction types

First, 132 pathways were obtained as *cancer-related pathways* for genes in the KEGG '*Pathways in cancer'* using online tool DAVID[17] (Supplementary Data 3). Then, for the paired agents, each could be mapped to *cancer-related pathways* to which its target proteins belong using DAVID, and two pathway sets were thereby constructed for these two agents. The relation between a pair of pathways that come from two pathway sets could be grouped into one of the four categories:

1. *Identical pathway pair*: The two pathway come from the same *cancer-related pathway set* (with the same pathway entry ID).

2. *Cross-talking pathway pair*: those pathways which are not identical, but sharing at least one common gene between the two *cancer-related pathway sets*.

3. *Interacting pathway pair*: those pathway which are neither identical nor cross-talking, but having genes with PPI interactions (via online PPI databases[10, 11, 12, 13]) from the two *cancer-related pathway sets*.

4. *Unrelated pathway pair*: those pathways which cannot be classified into any of above 3.

For a given drug pair x and y, the four types of pathway categories are calculated as below::

$$MP.I = \frac{Identical(x,y)}{\mathrm{M}\times\mathrm{N}}$$

$$MP.C = \frac{Crosstalking(x,y)}{\mathrm{M}\times\mathrm{N}}$$

$$MP.Int = \frac{Interacting(x,y)}{\mathrm{M}\times\mathrm{N}}$$

$$MP.U = \frac{Unrelated(x,y)}{\mathrm{M}\times\mathrm{N}}$$

Identical (*x*, *y*), *Crosstalking* (*x*, *y*), *Interacting* (*x*, *y*), and *Unrelated* (*x*, *y*) is the number of

identical pathways, crosstalking pathways, interacting pathways and unrelated pathways between pathway pair in the two pathway sets for drugs. M, N is the number of pathways in each pathway set.

The known synergetic agent pairs were found to tend to target the unrelated pathway pairs (*Z-score*=3.22) compared with the background pairs.

Supplementary Note 12: Manifold ranking method

A well-defined semi-supervised learning method incorporating a manifold ranking algorithm (Dengyong Zhou, et al, 2004) was applied in our study to build the preliminary ranking model of RACS.

The overview of the ranking model:

Given a set of samples: $X = \{x_1, \ldots x_q, x_{q+1}, \ldots, x_n\} \subset R^m$, the first q points are known synergistic drug pairs and the rest are unlabeled ones waiting to be ranked according to their relevance to the positive samples . In our study we have the 7-dimensional feature vector to represent the individual drug pair. Euclidean distance $d(x_i, x_j)$ can be derived for two drug pairs based on the 7-D feature vector.

Then let d: $X \times X \rightarrow R$ denotes a metric of Euclidean distance on the drug pair sample set X. Let f: $X \rightarrow R$ denote a ranking function which assigns to each pair x_i a ranking value f_i. f can be viewed as a vector $f = [f_1, \ldots, f_n]^T$. Let y: $X \rightarrow R$ denote an indicator function, in which $y_i = 1$ if x_i is the known synergistic pairs, and $y_i = 0$ otherwise. y can be viewed as $y = [y_1, \ldots, y_n]^T$. The goal of manifold ranking is to learn the ranking function f, which can be used to calculate the score to

rank the unlabeled (n-q+1) drug pairs.

Following is the detailed algorithm (Dengyong Zhou, et al, 2004):

1. Sort the pairwise distances among pairs in ascending order. Repeat connecting the two drug pairs with an edge according the order until a connected graph is obtained.
2. Form the similarity matrix W defined by $W_{ij} = 1/d(x_i, x_j)$ if there is an edge linking x_i and x_j. Note that $Wii = 0$ because there are no loops in the graph.
3. Symmetrically normalize W by $S = D^{-1/2}WD^{-1/2}$ in which D is the diagonal matrix with (i, i)-element equal to the sum of the i-th row of W.
4. Iterate $f(t + 1) = \alpha Sf(t) + (1-\alpha)y$ until convergence, where α is a parameter in [0, 1). In our study, $\alpha = 0.9$.
5. Let fi^* denote the limit of the sequence $\{f_i(t)\}$. Rank each drug pair x_i according its ranking scores fi^* (largest ranked first). This can be taken as the final ranking list of the drug pairs waiting to be explored.

The intuitive explanations of this algorithm are summarized in the following: (1) Euclidean distance *matrix* can be derived for each two drug pairs based on the 7-D feature vector. This distance matrix can be further transformed into a similarity matrix. (2) Initial ranking scores of positive pairs are distributed to those unknown samples based on similarity matrix. (3) Above step is repeated until a global stable state is achieved for ranking scores as an output. The core idea of this manifold ranking model is to rank the samples according to the global similarity collectively revealed by not only the positive but also the unlabeled data. The advantage of such model is to deal with cases with only limited positive/labelled samples.

Supplementary Note 13: Secondary filtering system

***Array data for Positive/labelled samples*:** Gene expression profiles for individual drugs in positive samples were searched from CMAP[18], ArrayExpress[19], and GEO[20]. As these synergistic drug combinations were indicated for different cancer types (Supplementary Fig. 20), gene expression profiles on the same cell line of the indicated cancer type were searched for each paired drugs. Finally, gene expression profiles for 9 pairs of the positive pair samples (including 11 drugs) were found (Supplementary Table 7).

***Array data for human β-cell lymphoma cell line Ly3*:** These gene expression data treated by individual drugs in human β-cell lymphoma cell line Ly3 were downloaded from DREAM website.

***Array data for lung adenocarcinoma cell A549 and ER positive breast cancer cell MCF7*:** Gene expression profiles for the testing drugs for lung cancer A549 cell line and breast cancer MCF7 cell line were also searched from CMAP, ArrayExpress, and GEO.

For each drug, the dataset was classified into a control group and a drug-treated group, each with no less than 3 samples. *T*-test was performed on the expression values of each gene in the two sample groups. Only genes with $P\_value < 0.05$ were used as the differentially expressed genes (DEGs).

2-step significance test

Take one parameter of DEG_Overlap as example. We have 9 positive samples and 46 unlabeled samples. For each drug pair, a group of DEG can be identified for each drug and DEG_Overlap can be derived for each drug pair. Step-1 is to test whether the two DEG sets are randomly overlapping or not for a drug pair. A p-value can be derived for each drug pair. While

Step-2 is to test whether those drug pairs with p-value < 0.05 of DEG_Overlap are enriched in the positive sample sets.

Step-1: To test whether the two DEG sets are randomly overlapping, DEG_Overlap should be compared to the overlapping rate of randomly selected gene sets for the same drug pair. For each drug of a pair, the same number of DEG sets was randomly chosen from the whole gene set in Affymetrix Microarray HGU133-A (11,961 genes). The DEG_Overlap was then calculated between the two simulated genes sets for 10,000 times. In this way, a p-value can be obtained as the probability of getting a simulated value of DEG_Overlap bigger than the actual DEG_Overlap of this drug pair, as synergistic drug pairs was reported with higher overlapping DEG than non-synergistic pairs[21].

Step-2: A hypergeometric test was adopted to test whether those drug pairs with p-value < 0.05 are enriched or not in the positive sample sets comparing to unlabeled sample sets. In step 1, a p-value was calculated for each pair (Supplementary Fig. 21).

$$P = 1 - \sum_{i=0}^{k-1} f(i) = 1 - \sum_{i=0}^{k-1} \frac{\binom{K}{i}\binom{N-K}{n-i}}{\binom{N}{n}}$$

where N is the number of samples in whole testing set, n is the number of pairs with significant p-value (p<0.05) in whole testing samples; K is the number of positive samples, while k is the number of positive pairs with significant p-value (p<0.05). Here it assumes that all the individually sampling events are independent.

In this step, we found that for both features of DEG_Overlap and Pathway_Coverage, drug pairs with statistically significant p-values (p<0.05) were enriched in positive samples (Supplementary Table 3)."

Supplementary References:

1. Larry M. Manevitz, Yousef M. One-Class SVMs for Document Classification. *Journal of Machine Learning Research* **2**, 139-154 (2001).

2. Barretina J*, et al.* The Cancer Cell Line Encyclopedia enables predictive modelling of anticancer drug sensitivity. *Nature* **483**, 603-607 (2012).

3. Carmichael J, DeGraff WG, Gazdar AF, Minna JD, Mitchell JB. Evaluation of a tetrazolium-based semiautomated colorimetric assay: assessment of radiosensitivity. *Cancer research* **47**, 943-946 (1987).

4. Chou TC, Talalay P. Quantitative analysis of dose-effect relationships: the combined effects of multiple drugs or enzyme inhibitors. *Advances in enzyme regulation* **22**, 27-55 (1984).

5. Hou W*, et al.* Synergistic antitumor effects of liposomal honokiol combined with adriamycin in breast cancer models. *Phytotherapy research : PTR* **22**, 1125-1132 (2008).

6. Westerfield M. *The zebrafish book: a guide for the laboratory use of zebrafish Danio (Brachydanio) rerio* (1995).

7. Kimmel CB, Ballard WW, Kimmel SR, Ullmann B, Schilling TF. Stages of embryonic development of the zebrafish. *Developmental dynamics : an official publication of the American Association of Anatomists* **203**, 253-310 (1995).

8. Lawson ND, Weinstein BM. In vivo imaging of embryonic vascular development using transgenic zebrafish. *Developmental biology* **248**, 307-318 (2002).

9. White RM*, et al.* Transparent adult zebrafish as a tool for in vivo transplantation analysis. *Cell stem cell* **2**, 183-189 (2008).

10. Keshava Prasad TS*, et al.* Human Protein Reference Database--2009 update. *Nucleic acids research* **37**, D767-772 (2009).

11. Chatr-aryamontri A*, et al.* MINT: the Molecular INTeraction database. *Nucleic acids research* **35**, D572-574 (2007).

12. Hermjakob H*, et al.* IntAct: an open source molecular interaction database. *Nucleic acids research* **32**, D452-455 (2004).

13. Stark C, Breitkreutz BJ, Reguly T, Boucher L, Breitkreutz A, Tyers M. BioGRID: a general repository for interaction datasets. *Nucleic acids research* **34**, D535-539 (2006).

14. Xenarios I, Salwinski L, Duan XJ, Higney P, Kim SM, Eisenberg D. DIP, the Database of

Interacting Proteins: a research tool for studying cellular networks of protein interactions. *Nucleic acids research* **30**, 303-305 (2002).

15. Pagel P*, et al.* The MIPS mammalian protein-protein interaction database. *Bioinformatics* **21**, 832-834 (2005).

16. Kanehisa M, Goto S, Sato Y, Furumichi M, Tanabe M. KEGG for integration and interpretation of large-scale molecular data sets. *Nucleic acids research* **40**, D109-114 (2012).

17. Huang da W, Sherman BT, Lempicki RA. Systematic and integrative analysis of large gene lists using DAVID bioinformatics resources. *Nature protocols* **4**, 44-57 (2009).

18. Lamb J*, et al.* The Connectivity Map: using gene-expression signatures to connect small molecules, genes, and disease. *Science* **313**, 1929-1935 (2006).

19. Brazma A*, et al.* ArrayExpress--a public repository for microarray gene expression data at the EBI. *Nucleic acids research* **31**, 68-71 (2003).

20. Edgar R, Domrachev M, Lash AE. Gene Expression Omnibus: NCBI gene expression and hybridization array data repository. *Nucleic acids research* **30**, 207-210 (2002).

21. Zhao J, Zhang XS, Zhang S. Predicting cooperative drug effects through the quantitative cellular profiling of response to individual drugs. *CPT: pharmacometrics & systems pharmacology* **3**, e102 (2014).
